# The DEAD-box protein Dbp6 is an ATPase and RNA annealase interacting with the peptidyl transferase center (PTC) of the ribosome

**DOI:** 10.1093/nar/gkac1196

**Published:** 2023-01-05

**Authors:** Ali Khreiss, Régine Capeyrou, Simon Lebaron, Benjamin Albert, Katherine E Bohnsack, Markus T Bohnsack, Yves Henry, Anthony K Henras, Odile Humbert

**Affiliations:** Molecular, Cellular and Developmental Biology Unit (MCD), Centre de Biologie Intégrative (CBI), Université de Toulouse, CNRS, UPS, 31062 Toulouse, France; Molecular, Cellular and Developmental Biology Unit (MCD), Centre de Biologie Intégrative (CBI), Université de Toulouse, CNRS, UPS, 31062 Toulouse, France; Molecular, Cellular and Developmental Biology Unit (MCD), Centre de Biologie Intégrative (CBI), Université de Toulouse, CNRS, UPS, 31062 Toulouse, France; Molecular, Cellular and Developmental Biology Unit (MCD), Centre de Biologie Intégrative (CBI), Université de Toulouse, CNRS, UPS, 31062 Toulouse, France; Department of Molecular Biology, University Medical Center Göttingen, 37073 Göttingen, Germany; Department of Molecular Biology, University Medical Center Göttingen, 37073 Göttingen, Germany; Göttingen Center for Molecular Biosciences, Georg-August University Göttingen, 37077 Göttingen, Germany; Molecular, Cellular and Developmental Biology Unit (MCD), Centre de Biologie Intégrative (CBI), Université de Toulouse, CNRS, UPS, 31062 Toulouse, France; Molecular, Cellular and Developmental Biology Unit (MCD), Centre de Biologie Intégrative (CBI), Université de Toulouse, CNRS, UPS, 31062 Toulouse, France; Molecular, Cellular and Developmental Biology Unit (MCD), Centre de Biologie Intégrative (CBI), Université de Toulouse, CNRS, UPS, 31062 Toulouse, France

## Abstract

Ribosomes are ribozymes, hence correct folding of the rRNAs during ribosome biogenesis is crucial to ensure catalytic activity. RNA helicases, which can modulate RNA–RNA and RNA/protein interactions, are proposed to participate in rRNA tridimensional folding. Here, we analyze the biochemical properties of Dbp6, a DEAD-box RNA helicase required for the conversion of the initial 90S pre-ribosomal particle into the first pre-60S particle. We demonstrate that *in vitro*, Dbp6 shows ATPase as well as annealing and clamping activities negatively regulated by ATP. Mutations in Dbp6 core motifs involved in ATP binding and ATP hydrolysis are lethal and impair Dbp6 ATPase activity but increase its RNA binding and RNA annealing activities. These data suggest that correct regulation of these activities is important for Dbp6 function *in vivo*. Using *in vivo* cross-linking (CRAC) experiments, we show that Dbp6 interacts with 25S rRNA sequences located in the 5′ domain I and in the peptidyl transferase center (PTC), and also crosslinks to snoRNAs hybridizing to the immature PTC. We propose that the ATPase and RNA clamping/annealing activities of Dbp6 modulate interactions of snoRNAs with the immature PTC and/or contribute directly to the folding of this region.

## INTRODUCTION

The eukaryotic cytoplasmic ribosome, responsible for protein synthesis, is a complex ribonucleoprotein particle (RNP) consisting of a small and a large subunit, respectively composed of the 18S ribosomal RNA (rRNA) and the 5S, 5.8S and 25S/28S (yeast/mammals) rRNAs associated with ribosomal proteins (RPs). Ribosomes are ribozymes as their catalytic activity is mediated by specific rRNA domains. For these domains to be functional, the rRNAs must be correctly folded and compacted. One of the key aspects of ribosome biogenesis is, therefore, to convert the nascent rRNA precursor into highly organized and precisely folded mature rRNA molecules thus generating a functional architecture competent for translation. In yeast, production of the two ribosomal subunits is the result of successive maturation steps of a long nascent precursor transcript (35S pre-rRNA), encompassing the sequences of the 18S, 5.8S and 25S rRNAs (1–3). Maturation of this pre-rRNA takes place in the context of pre-ribosomal particles and comprises pre-rRNA nucleotide modifications, cleavages, and folding events concomitant with the progressive association with RPs. In addition to 75 small nucleolar (sno)RNPs involved in nucleotide modifications and pre-rRNA folding, >200 assembly factors (AFs) participate in the maturation steps leading to the large and the small subunits. They interact transiently with pre-ribosomal particles but are not present in the mature subunits. These factors include ribonucleases, RNA modifying enzymes, GTPases, ATPases, protein kinases and phosphatases, and members of the so-called ‘RNA helicase’ family (1,4,5). The latter factors form the most prominent group of enzymatic AFs and are mainly DEAD-box proteins (6). In *Saccharomyces cerevisiae*, the vast majority of RNA helicases acting on pre-ribosomal particles are essential for growth (7,8). They are anticipated to help the rRNAs reach and maintain their functional conformational states in a competitive process controlled by equilibrium dynamics. Some have been shown to be involved in the regulation of snoRNA/pre-rRNA interactions (9–16). It is also widely accepted that RNA helicase family members play important roles in regulating the folding of pre-rRNAs, although experimental proof of this is limited.

The name ‘helicase’ was coined because members of the family, defined on the basis of sequence homology (17), were initially found to be able to unwind base-paired nucleic acid strands. This term is, however, a misnomer, as some members do not display such unwinding activity, at least *in vitro* (18). RNA helicases all share the ability to bind to and hydrolyze NTPs (usually ATP) and to bind to nucleic acids (19). Hence, RNA helicases can contribute to the remodeling of RNA secondary structures because they bind to RNA/RNA duplexes as well as RNA/protein complexes and modulate RNA/RNA and RNA/protein interactions in an NTP-dependent manner. Another function of RNA helicases is to serve as RNA anchors within macromolecular RNP complexes. Such helicases act as bridges between the RNA and protein components of the complex, a phenomenon generally termed ‘clamping’. Well studied examples of enzymes with these properties include the RNA helicases eIF4A-III, Mss116 and Ded1 (20–22). Numerous studies have led to structural models for DEAD-box protein family members (18,19). Typical DEAD box RNA helicases contain a helicase core formed by two RecA-like domains connected by a short flexible linker and conserved sequence motifs are distributed along the RecA-like domains. The DEAD-box protein sub-family is named after the amino acid sequence of the conserved core motif II. Conserved motifs are involved in RNA binding, ATP binding and hydrolysis, and the coordination of ATP and RNA binding. The two major ATP binding motifs (I and II) are located in RecA-1 domain and the RecA-1 and RecA-2 domains can form a pocket in which ATP is lodged. In the absence of ligands, the helicase core is in an open conformation, where the two RecA domains can move independently of one another. Upon binding of either ATP or RNA, these two domains get closer to each other, leading to core closure promoting conformational changes. In addition to the common core, RNA helicases frequently contain flanking regions that fulfill diverse functions, such as modulating nucleotide binding and hydrolysis, conferring substrate specificity, and/or binding to accessory proteins. The DEAD-box proteins form the largest group of RNA helicases and they appear to be extremely similar in terms of structural features. DEAD-box proteins typically operate through a non-directional and non-processive mechanism. As described in several structural studies reporting co-crystallization of DEAD-box helicases with synthetic RNA oligonucleotides, the proteins interact with the sugar-phosphate backbone of the RNA molecule (23–27). As nucleoside bases are not involved in the interaction, DEAD-box helicases are expected to interact with RNA in a sequence-independent manner. Many of these proteins function in cooperation with protein cofactors that regulate their biochemical activities and/or recruit them to their RNA targets (28).

The early steps of large ribosomal subunit synthesis involve numerous RNA helicases. Indeed, no less than seven RNA helicases, namely Mak5, Has1, Prp43, Dbp3, Dbp7, Dbp9 and Dbp6 are required for production of the earliest pre-60S particles (7,11,29–32). These RNA helicases likely drive the initial pre-rRNA folding and assembly events necessary for the production of early pre-60S particles. These events remain poorly understood despite their paramount importance for the synthesis of the large ribosomal subunit. The few available data concerning the first pre-60S particle indicate that some key determinants of rRNA folding are already established in this particle (31,33). It is envisaged that failure to correctly complete these early folding events abrogates production of the first pre-60S particle, thus compromising large ribosomal subunit formation (34). Among the six RNA helicases that participate in the initial structural steps, the DEAD-box protein Dbp6 (35) belongs to a protein complex containing four additional AFs, Npa1, Npa2, Nop8 and Rsa3 (30,36). The absence of Dbp6, or any other protein of the complex, inhibits production of early pre-60S particles. To date, however, the importance of Dbp6 catalytic activities for 60S subunit synthesis has not been assessed nor have the RNA substrates of Dbp6 been identified. Here, we analyze the biochemical properties of Dbp6 and assess the effects of targeted Dbp6 amino acid substitutions that impair these activities on ribosome biogenesis. Our results show that Dbp6 binds to and hydrolyses ATP *in vitro*. Moreover, Dbp6 is endowed with an RNA annealing activity and binds stably to the resulting RNA duplex. Substitution of conserved residues of Dbp6 in motifs I and II does not preclude incorporation of the mutant proteins into 90S pre-ribosomal particles but impairs their function in 60S subunit synthesis. *In vivo* RNA-protein cross-linking analyses indicate that Dbp6 binds to 25S rRNA sequences that will form the peptidyl transferase center (PTC) as well as snoRNAs that guide nucleotide modifications in the PTC region. Taken together, we propose that Dbp6 ATPase and RNA annealing activities contribute to remodeling events in the PTC region during the early stages of pre-60S particle assembly.

## MATERIALS AND METHODS

### *Escherichia coli* strains and plasmids

pET-Dbp6 was obtained by cloning into a modified pET-15b (Novagen) plasmid in the NdeI and BamHI restriction sites a *DBP6* ORF PCR cassette downstream of the polymerase T7 promoter and a His tag (His_6_) by the Ligation Independent Cloning (LIC) method using oligonucleotides listed in Supplementary Table S1. *DBP6* mutants were generated by reverse PCR with In-Fusion® HD Cloning Plus (Takara/Clontech) using primers listed in Supplementary Table S1 and the pET-Dbp6 plasmid as template. The PCR products were transformed into *Escherichia coli* Stellar cells (Takara), and plasmids from clones into Rosetta λDE3 strain to produce pET-MII/MI/MIa/MIc or ΔN plasmids.

### Yeast strains and media

The GAL::HA-DBP6 (36) strain expressing the HA-tagged ORF of Dbp6 under the control of the *GAL1-10* promoter has been transformed with plasmids pHA113/WT/MII/MI/MIa/MIc or ΔN. These plasmids were obtained by cloning into the pHA113 plasmid (37) in the BamHI restriction site, in between a *GAR1* promoter and a ZZ tag, a *DBP6* WT/MII/MI/MIa/MIc or ΔN cassette amplified by PCR using the pET-MII/MI/MIa/MIc or ΔN plasmids (Supplementary Tables S4).

A strain expressing wild type Dbp6 tagged with HTP (His tag-Tev cleavage site-ZZ sequence derived from *Staphylococcus aureus* Protein A) has been obtained by transforming the GAL::HA-DBP6 strain with a plasmid derived from pRS415 (38) containing a *DBP6* WT cassette (Supplementary Tables S4).

Strains have been grown in YP (1% yeast extract, 1% peptone) supplemented with either 2% galactose or 2% glucose or YNB (1.7 g/l) supplemented with ammonium sulphate (5 g/l) and 2% galactose or 2% glucose.

### Oligonucleotides

RNA/DNA oligonucleotides are listed in Supplementary Tables S1 (primers for generating Dbp6 mutants), S2 (oligonucleotides for *in vitro* assays) and S3 (northern blot probes). When necessary, the oligonucleotides were radiolabeled using T4 PNK enzyme (Promega) and 25 μCi [γ-^32^P]-ATP (PerkinElmer) per probe.

### Purification of his-tagged recombinant proteins

*E. coli* Rosetta λDE3 bacteria transformed with plasmids pET-Dbp6/MII/MI/MIa/MIc or ΔN, were grown at 37°C in 2× YT medium supplemented with 100 μg/ml ampicillin and 25 μg/ml chloramphenicol to an OD_600nm_ ∼0.5. Isopropyl-β-d-thiogalactopyranoside (IPTG) was then added to a final concentration of 0.5 mM and culturing was continued overnight (∼15 h) at 18°C. The cells were harvested by centrifugation, washed with 1× PBS and then dry frozen at –80°C. Bacteria were thawed, resuspended in lysis buffer (50 mM Tris–HCl pH 7.4, 500 mM NaCl, 10% glycerol, 1 mM DiThioThreitol (DTT), 0.5 mM phenylmethylsulfonyl fluoride (PMSF) supplemented with EDTA-free protease inhibitors (Roche)) and lysozyme was added to a final concentration of 150 μg/ml. The cell suspension was sonicated on ice and then subjected to affinity chromatography using a HisTrap FF column (GE Healthcare). His-tagged proteins were eluted with a linear gradient of imidazole. The eluted fractions containing His-tagged proteins were pooled and the proteins of interest were purified by size exclusion chromatography with a Superdex 200 gel filtration column (GE Healthcare) in GF-ATPase buffer (20 mM Tris–HCl pH 8.0, 100 mM KCl, 10% glycerol, 1 mM DTT and 0.5 mM PMSF). The integrity of purified His-tagged proteins was checked by PAGE followed by Coomassie blue staining and proteins were stored at –80°C. The identity of the purified polypeptides (WT and mutant) of the size expected for Dbp6 was further checked by mass spectrometry. *In vitro* assays were repeated with different batches of purified proteins.

### Immunoprecipitation experiments

Immunoprecipitation experiments were performed as described before (36). Protein samples were analyzed by western blot and RNA samples by northern blot, as described below.

### RNA extractions and northern blot analyses

Yeast total RNAs were extracted as described (39). In all northern blot experiments, equal amounts of total RNAs (3 μg) were analyzed. RNAs were separated as described in ‘Molecular Cloning’, Sambrook and Russel, CSHL Press (‘Separation of RNA According to Size: Electrophoresis of Glyoxylated RNA through Agarose Gel’). RNAs were transferred to Amersham Hybond N+ membranes (GE Healthcare) which were hybridized with ^32^P-labeled oligonucleotide probes using Rapid-hyb buffer (GE Healthcare). Radioactive membranes were exposed to Phosphorimaging screens and revealed using a Typhoon Tryo (GE Healthcare). The signals corresponding to the pre-rRNAs were quantified using the ImageQuant software (Molecular Dynamics). Sequences of the oligonucleotides used as probes in this study are listed in Supplementary Table S3.

### Western blot

Proteins were separated by electrophoresis on 8% or 10% 29:1 polyacrylamide gels in 1× TGS buffer (25 mM Tris, 192 mM glycine, 0.1% SDS, pH 8.3) and transferred onto nitrocellulose membranes using the Bio-Rad TransBlot system. Membranes were saturated by 30 min incubation in TBS-T buffer (Tris-buffered saline with 0.1% Tween 20), containing 5% (w/v) powder milk. Specific proteins were detected by incubation in TBS-T with the following antibodies: anti-HA-HRP (Clone 3F10, Roche) diluted 1000-fold; anti-Dbp6 polyclonal antibodies diluted 10 000-fold (36); anti-PGK1 (clone 22C5D8, Invitrogen) diluted 10 000-fold; rabbit peroxidase anti-peroxidase soluble complex (PAP, Sigma) diluted 10 000-fold. After incubation with primary antibodies, membranes were washed three times with TBS-T. Anti-rabbit or mouse IgG–HRP conjugate (Promega) were used, when needed, as secondary antibodies. After three washes of the membranes with TBS-T, proteins associated with antibodies were detected as chemiluminescent signals using Clarity Western ECL Substrate (Bio-Rad) and the ChemiDoc imager apparatus (Bio-Rad) followed by quantification with the ImageLab software (Bio-Rad).

### Protein-protein cross-linking

Following incubation in 5 μl of RB 1× reaction buffer (2.5 mM Tris–HCl pH 8, 2.5 mM MgCl_2_, 10 mM KCl, 0.02 mM DTT, 10 mg/ml BSA) for 15 min at 20°C, purified recombinant Dbp6 (1.5 or 0.8 μM) was placed in drops on an open petri dish and cross-linked by exposure at 1 cm to a UV_312 nm_ lamp (UVItec Limited) for 6 min, on ice. Samples were then diluted twice in H_2_O and proteins were separated by SDS-PAGE on 6% (37.5:1) gels. Proteins were analyzed either by western blot or silver staining (SilverQuest™, Invitrogen).

### ATP binding assay

ATP cross-linking experiments were performed following the same procedure as described above (‘protein-protein cross-linking’), except that 0.2 μM [α-^32^P]-ATP (PerkinElmer) and cold ATP (1 or 5 μM, as indicated) were added to the reaction.

### ATPase assay

Dbp6 proteins (0.4–1 μM) were incubated in a reaction mix containing RB 1× (see above), 100 μM cold ATP and 0.6 μCi/μl [α-^32^P]-ATP (PerkinElmer) with or without 2 μM of ss58-mer oligonucleotide RNA. The reaction mix was incubated at 30°C and 1 μl aliquots taken from the mix at the appropriate time points were loaded on a Polygram CEL 300PEI/UV254 (Macherey-Nagel™) chromatography plate. Chromatography was performed in 0.75 M KPO_4_ buffer. The radioactive plate was dried and signals corresponding to [α-^32^P]-ATP and its hydrolysis product [α-^32^P]-ADP were revealed by Phosphorimager (Typhoon Tryo, GE Healthcare) and quantified using the ImageQuant software (Molecular Dynamics).

### RNA binding assay

The protein at a fixed or a range of concentrations was mixed with 1 nM of radiolabeled ss38-mer or ds38/58 substrate, RB 1× (see above) and 0.03 units RNasin. The reaction was incubated for 15 min at 30°C and 2 μl of specific loading buffer (50% glycerol, 0.01% xylene cyanol and 1 mg/ml BSA) was added by short spin centrifugation. Samples were separated on a 37.5:1 polyacrylamide TBE gel run at 120 V for 35 min. After drying the gel, the radioactive signals were revealed by Phosphorimager (Typhoon Tryo, GE Healthcare), and quantified using the ImageQuant software (Molecular Dynamics).

The ds38/58 substrate was produced as follows: the two oligonucleotides, ss38-mer and ss58-mer, were mixed in equimolar amounts in a 10 mM Tris–HCl pH7.5, 1 mM EDTA and 100 mM NaCl buffer, incubated 5 min at 85°C and transferred quickly on ice.

### Unwinding and annealing assays

**Figure 1.**
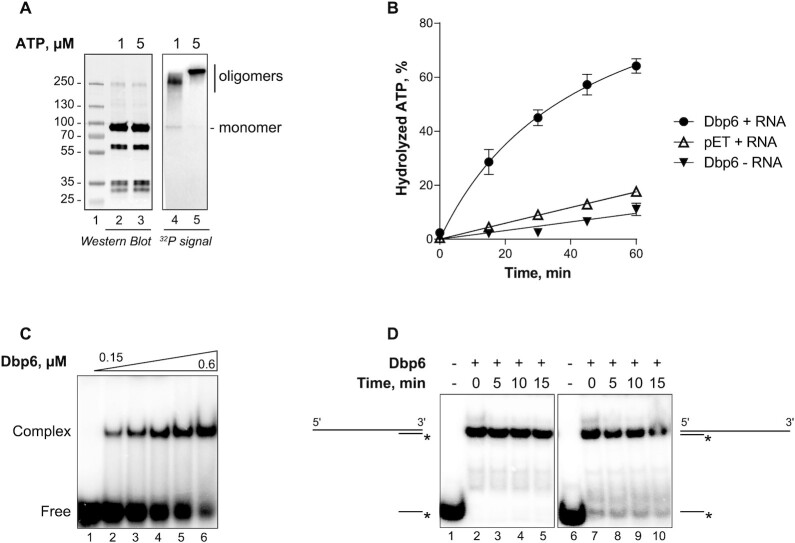
Biochemical properties of the Dbp6 protein. (**A**) UV cross-linking assays to detect Dbp6 binding to radioactive [α^32^P]-ATP in the presence of an excess of unlabeled ATP, 1 or 5 μM as indicated. Protein species, monomers or oligomers as indicated, were visualized by western blot using anti-Dbp6 antibodies (lanes 2 and 3). The radioactive ATP-Dbp6 complexes were revealed by phosphorimaging (lanes 4 and 5). Lane 1: pre-stained protein marker. (**B**) ATPase activity, monitored by the percentage of ATP hydrolyzed from initial 100 μM ATP as a function of time, obtained with purified Dbp6 (Dbp6, 0.8 μM), or a mock purification fraction (pET), both in presence of RNA (2 μM), or Dbp6 in absence of RNA (–RNA). The same volumes of the same size exclusion chromatography fractions from the Dbp6 or mock (pET) purifications were used. Data points are averages from multiple measurements (*n* ≥ 3), error bars represent the standard error of the mean (SEM). (**C**) RNA binding activity of Dbp6 tested on radiolabeled RNA oligonucleotide (ss38-mer). Representative gel shows formation of complexes on substrate with increasing concentration of protein as follows: 0, 0.15, 0.225, 0.3, 0.45, 0.6 μM. (**D**) Unwinding activity of Dbp6 (1 μM) was tested on two substrates with 5′ or 3′ single-stranded overhangs (left and right panels, respectively) in time course reactions performed in the presence of ATP (1 mM). The substrates were formed by hybridization of a 113 nucleotide-long RNA (113 ssRNA, Supplementary Table S2) to a short 21 nucleotide-long oligonucleotide at either the 3′ or 5′ extremity (either Comp-3′ or Comp-5′, Supplementary Table S2). After proteinase K treatment of the samples, the RNA species were separated by non-denaturing PAGE. The hemi-duplexes and free oligonucleotides corresponding to the substrates and products of the unwinding reactions, respectively, are schematized. Lanes 1 and 6 were loaded with isolated labeled Comp-3′ or Comp-5′ oligonucleotides, respectively.

**Figure 2.**
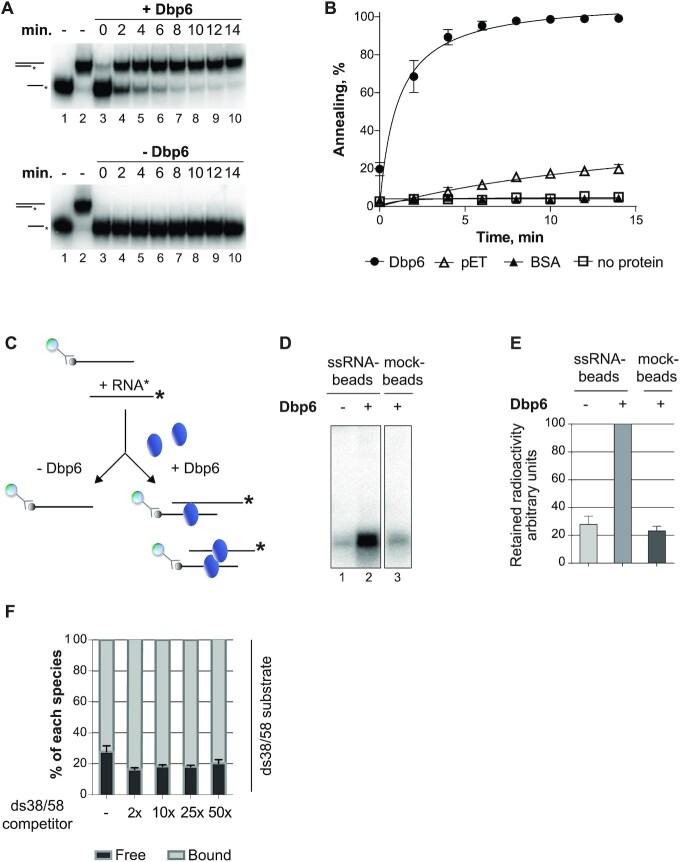
Annealing activity of the Dbp6 protein. (**A**) Stimulation of annealing of two complementary oligonucleotides by Dbp6. The annealing reactions were performed, in 10 μl at 30°C, by incubating for the indicated times two complementary oligonucleotides of different lengths, ss58-mer and radiolabeled ss38-mer, with 0.25 μM (upper panel) or without (lower panel) Dbp6. No ATP was added to the reactions. The deproteinized products of the reaction (hemi-duplex and free oligonucleotide, schematized on the left of the gel) were separated by non-denaturing PAGE and visualized by phosphorimaging. Lanes 1 and 2 correspond to the free oligonucleotide and hemi-duplex as markers, respectively. (**B**) Percentage of annealing calculated from quantification of annealed RNA products in the presence of either (0.5 μM) Dbp6 protein (Dbp6), mock purification fraction (pET), BSA (BSA), or without protein (no protein). (**C**) Scheme for the RNA bridging reaction. A biotinylated oligonucleotide (ss38-mer) is mixed with a non-complementary radiolabeled trans-oligonucleotide (non-biotinylated ss38-mer) in the presence or absence of Dbp6. After removing the supernatant, the material retained on streptavidin beads is treated with proteinase K, and the presence of radioactive oligonucleotide is detected by non-denaturating PAGE followed by phosphorimaging. (**D**) Representative scan of the non-denaturing gel loaded with products of the bridging reactions. A radioactive band corresponding to the trans-oligonucleotide is enriched on the beads in the presence of the Dbp6 protein (lane 2), compared to the absence of Dbp6 (lane 1) or the absence of biotinylated RNA on the beads (lane 3). (**E**) Quantification of the radiolabeled trans-oligonucleotide retained on the beads. The quantity retained on the ssRNA-beads in the presence of Dbp6 is arbitrarily set at 100. (**F**) Stability of Dbp6 binding on hemi-duplex challenged by competitors. Electrophoretic mobility shift assays were performed with Dbp6 and the radiolabeled ds38/58 substrate (ss38-mer hybridized to ss58-mer) in the presence of increasing amounts of the same, but non-radioactive, ds38/58 substrate, indicated as fold-excess compared to the radiolabeled substrate. The histogram shows the quantification of each labeled species (free or bound). Error bars in all cases correspond to SEM for *n* ≥ 3 experiments.

For the unwinding assays shown in Figure 1D, substrates (1 nM) with 5′ or 3′ overhangs, formed by hybridization of a 113 nucleotide-long ssRNA (113 ssRNA, (40) to radiolabeled 21 nucleotide-long Comp-3′ or Comp-5′, were incubated with Dbp6 protein (0.5–1 μM) in RB 1× (see above) and 0.03 units RNasin, supplemented with 1 mM of ATP, at 30°C. At different times, the reaction was stopped with Stop buffer (5% glycerol, 0.2% SDS, 0.2 mg/ml proteinase K, 2 mM Tris–HCl pH 7.5, 0.01% xylene cyanol, 0.01% bromophenol blue) containing 2 μM of cold trap oligonucleotide (either Comp-5′ or Comp-3′) to prevent reannealing of the radiolabeled Comp-5′ or Comp-3′ oligonucleotides to the 113 ssRNA. For the annealing assays, Dbp6 protein (0.25 to 0.025 μM) was mixed with 1 nM of radiolabeled ss38-mer RNA oligonucleotide, 1 nM of cold ss58-mer RNA oligonucleotide, 0.03 units RNasin (Promega) in RB 1× in a final volume of 10 μl. A time-course reaction was performed at 30°C and stopped with Stop buffer containing 6.25 nM trap oligonucleotides (Comp-58 and Comp-3′) that are complementary to the ss58-mer and used to prevent annealing of the radiolabeled ss38-mer oligonucleotide. The ds38/58 marker loaded in lane 2 of Figure 2A was produced as follows: the radiolabeled ss38-mer and the unlabeled ss58-mer oligonucleotides were mixed in equimolar amounts in a buffer containing 10 mM Tris–HCl pH 7.5, 1 mM EDTA and 100 mM NaCl, incubated 5 min at 85°C and transferred quickly on ice. For both unwinding and annealing assays, ssRNAs and hemi-duplexes were detected, after migration on 19:1 polyacrylamide TBE gel, by exposure of the dried gel to a phosphorimager screen and scan on a Typhoon Tryo (GE Healthcare). Ratios of annealed and free RNA oligonucleotides were calculated from quantifications with ImageQuant software (Molecular Dynamics).

### Assay for RNA retention on RNA-beads

RNA-beads were prepared by mixing a biotinylated ss38-mer oligonucleotide with magnetic streptavidin-beads (Bio-Adembeads StreptaDivin, Ademtec) following instructions of the manufacturer. Free streptavidin binding sites were saturated by incubating RNA-beads with a large excess of biotin, followed by more washes. Mock-beads correspond to free biotin mixed with streptavidin-beads. For pull-down reactions, dried RNA-beads (equivalent to <2.5 pmol of RNA) were resuspended, or not, with 0.8 μM of Dbp6 protein in 20 μl RB 1× supplemented with 0.03 units RNasin (Promega) in the presence of radiolabeled trans RNA oligonucleotide (non-biotinylated ss38-mer, 0.0375 pmole) and incubated at 30°C for 15 min. Following incubation, RNA-beads were washed several times with RB 1×. The radiolabeled trans RNA retained on the RNA-beads was detected after resuspension of the dried beads with Stop buffer (5% glycerol, 0.2% SDS, 0.2 mg/ml proteinase K, 2 mM Tris–HCl pH 7.5, 0.01% xylene cyanol, 0.01% bromophenol blue) and electrophoresis on TBE acrylamide gel.

### UV cross-linking and analysis of cDNA (CRAC)

A plasmid derived from pRS415 (38) supporting expression of N-His_6_-TEV-Protein A (HTP) tagged Dbp6 was transformed into the GAL::HA-DBP6 strain. CRAC experiments were performed as previously described (36,38). After a two-step Dbp6 purification and partial digestion of bound RNAs, the miRCat-33 linker (5′-AppTGG AAT TCT CGG GTG CCA AG/ddC/-3′) was ligated to the 3′ end of the RNAs on the Ni-NTA beads with 800 units of T4 RNA ligase 2 truncated K227Q (New England Biolabs) in 1× PNK buffer/16.67% PEG 8000 in the presence of RNasin. The 5′ ends of the RNAs were then radiolabeled by phosphorylation in reactions containing 1× PNK buffer, 40 μCi of ^32^P-γ ATP and 20 units of T4 PNK (Sigma) in a total volume of 80 μl. The reactions were incubated at 37°C for 40 min. To ensure all RNAs get phosphorylated at the 5′ end for downstream ligation of the 5′ linker, 1 μl of 100 mM ATP was added to the reaction mix, which was incubated for another 20 min at 37°C. Beads were washed four times with 1× PNK buffer. Solexa linkers L5Aa (5′-invddT-ACA CrGrAr CrGrCr UrCrUr UrCrCr GrArUr CrUrNr NrNrUr ArArG rC-OH-3′) and L5Ab (5′-invddT-ACA CrGrAr CrGrCr UrCrUr UrCrCr GrArUr CrUrNr NrNrAr UrUrAr GrC-OH-3′) were ligated to the 5′ end of the RNAs retained on the Ni-NTA beads for the BY4742 (control) and HTP-Dbp6 samples, respectively. Synthesis of cDNAs was performed using SuperScript III reverse transcriptase (Thermo Fisher Scientific) and oligonucleotide miRcatRT (5′-CCT TGG CAC CCG AGA ATT-3′). The resulting cDNAs were PCR-amplified using LA Taq DNA polymerase (TaKaRa) and primers P5F (5′-AAT GAT ACG GCG ACC ACC GAG ATC TAC ACT CTT TCC CTA CAC GAC GCT CTT CCG ATC T-3′) and P3R (5′-CAA GCA GAA GAC GGC ATA CGA GAT CCT TGG CAC CCG AGA ATT CC-3′). The resulting PCR products were purified by phenol:chloroform:isoamyl alcohol extraction and ethanol precipitation. After agarose gel electrophoresis (using agarose ‘small fragments’, Eurogentec) run in 1× TBE buffer and stained with SYBR Safe DNA gel stain (Invitrogen), DNA fragments ranging in size between 150 and 250 bp were gel purified using MinElute PCR Purification Kit (QIAGEN). Concentration of the final DNA samples was measured using Qubit™ dsDNA HS Assay Kit (Invitrogen) and a Qubit™ fluorometer (Thermo Fisher Scientific).

### Deep-sequencing and computational analyses

The cDNA samples were sent for Illumina MiSeq deep sequencing (EpiRNA-Seq facility, CNRS, Université de Lorraine, INSERM). Barcodes, adapters and low-quality reads were eliminated using Flexbar (http://sourceforge.net/projects/flexbar/). Remaining reads were aligned to the yeast genome using Novoalign (http://www.novocraft.com). Downstream analyses including the pileups were performed using the pyCRAC tool suite (http://sandergranneman.bio.ed.ac.uk/Granneman_Lab/pyCRAC_software.html). Hits repartition per million of sequences were produced using pyReadCounter.py-m 1 000 000 option. Different pileups of hits for each gene were obtained using pyPileup.py-L 50-limit = 100 000 options. NGS analysis files of raw and processed data were deposited in the Gene Expression Omnibus database under the accession number GSE200692 (For review only: secure token: udurgeoqprebrif).

## RESULTS

### Dbp6 exhibits biochemical properties of DEAD-box RNA helicases

In the first study describing Dbp6, sequence analysis revealed that Dbp6 belongs to the DEAD-box protein family of putative ATP-dependent RNA helicases (35). However, no experimental data assessing Dbp6 enzymatic activities have been reported. As a starting point for biochemical studies, recombinant His_6_-Dbp6 (*ca*. 73 kDa) was purified from *E. coli* by nickel affinity chromatography followed by size exclusion chromatography, to remove contaminating proteins. Dbp6 mainly eluted in a retention peak expected for a dimeric protein (elution volume of about 70.5 ml, 146 kDa, Supplementary Figure S1A). Based on the calibration of the column and on the theoretical molecular mass of the protein, the monomeric form was estimated to elute slightly later, in fractions corresponding to the downward slope of the curve (76 ml) (Supplementary Figure S1A). High molecular weight fractions (F5-F9, numbering indicated on the graph, Supplementary Figure S1A) as well as fractions from the main prominent peak (F14 to F18, Supplementary Figure S1A) were analyzed for the presence of Dbp6 by SDS-PAGE and Coomassie blue staining (Supplementary Figure S1B). The latter fractions were also analyzed by western blot using anti-Dbp6 antibodies (Supplementary Figure S1C). In the chromatogram, an additional small shoulder corresponding to proteins of smaller size was observed (elution volume of about 83 ml), which are truncated fragments of Dbp6 (Supplementary Figure S1C, lane 6). Although peak fractions F14 to F18 mostly contain full-length Dbp6, truncated fragments are also present, more so in fraction F18 compared to the others (Supplementary Figure S1B and C). To assess the relative amounts of monomer versus oligomers in the main peak fractions compared to high molecular weight fractions, we submitted aliquots of these fractions to UV cross-linking, then performed SDS-PAGE and western analysis or silver staining (Supplementary Figure S1D). Higher order oligomers are much more prevalent relative to the monomeric form in high molecular weight fraction 8 (Supplementary Figure S1D, lanes 4 and 8, ‘oligo’) than in fractions F17 and F18 (Supplementary Figure S1D, lanes 2, 3, 6 and 7). The observed oligomers formed by recombinant Dbp6 could be functionally relevant multimers, or instead may correspond to aggregated structures. Fraction F17 was utilized for further experiments as it likely contains mainly a monomeric form of the protein with minimal degradation.

As a member of the DEAD-box family, Dbp6 is expected to display the following biochemical properties: ATP binding and hydrolysis, RNA binding, and potentially RNA helicase activity. First, we examined the ability of the enzyme to bind to ATP *in vitro* by UV cross-linking radiolabeled nucleotides to the protein (Figure 1A). After a short (15 min) incubation of Dbp6 with a mixture of radiolabeled and unlabeled ATP in the absence of RNA, the presence of ATP bound to the protein was attested by radioactive signals on polyacrylamide gels at the position of Dbp6 oligomers (four- or five-order oligomers, lanes 4 and 5 respectively). Using high amounts of protein and long exposure times, a radioactive signal at the position of the monomers could also be detected (Figure 1A, lane 4). Western blot analysis of the protein profiles on native polyacrylamide gels (Figure 1A, lanes 2 and 3) revealed that the oligomers were barely detectable compared to the monomers. We conclude that Dbp6 binds to ATP *in vitro*. The low level of ATP-bound Dbp6 monomers obtained may be due to the fact that when ATP is bound to monomeric Dbp6, its cross-linking to the protein is very inefficient, perhaps because the positioning of the reactive functionalities is not well compatible with a cross-link.

We then determined whether Dbp6 was able to hydrolyze ATP by measuring its ATPase activity *in vitro*. Purified Dbp6 was incubated with radiolabeled [α-^32^P] ATP in the presence or absence of RNAs (ss58-mer oligoribonucleotide) and the percentage of hydrolyzed ATP was monitored over time by thin layer chromatography and PhosphorImager quantifications (Figure 1B). ATP hydrolysis increased in a time-dependent manner in the presence of RNA. This ATPase activity could be attributed to Dbp6 as a significantly lower ADP production was observed with a control fraction purified from *E. coli* cells carrying the empty expression vector in the presence of RNA (pET + RNA, Figure 1B). However, we cannot completely exclude that a minor fraction of this activity may be due to the presence of small amounts of contaminating *E. coli* ATPases, given the residual ATPase activity detected with the control fraction. Furthermore, we observed a similar basal ATPase activity when Dbp6 was incubated with ATP in the absence of RNA, indicating that Dbp6 behaves as an RNA-dependent ATPase. Although Dbp6 oligomers bind to ATP (Figure 1A), the size exclusion chromatography fraction containing Dbp6 oligomers did not display any ATPase activity (Supplementary Figure S1E), suggesting that they are deficient in ATP hydrolysis.

As the ATPase activity of Dbp6 depends on RNA, we next evaluated the RNA-binding properties of the enzyme. This was assessed using electrophoretic mobility shift assays (EMSAs). Dbp6 efficiently bound to a ^32^P-labeled single-stranded, 38 nucleotide RNA molecule, as attested by the appearance of a slow-migrating RNA-protein complex, which increased with the amount of Dbp6 protein added in the reaction (Figure 1C). As Dbp6 is involved in early steps of ribosome biogenesis where rRNA precursors and snoRNAs adopt highly dynamic structures, we tested the affinity of Dbp6 for different types of RNA substrates, either single-stranded, double-stranded or hemi-duplexes, and substrates of different lengths (Supplementary Figure S2A). Dbp6 bound to both single- and double-stranded RNAs. Comparing single- or double-stranded RNA molecules of a given length (either 58 or 38 nucleotides), the affinity was higher for the single-stranded RNAs compared to the double-stranded versions. Comparing RNAs of different lengths, the affinity was higher for the longer forms. Notably, no interaction was detected with the 21 nucleotide single-stranded substrate, suggesting that the minimal size of RNA substrates efficiently bound by Dbp6 is between 21 and 38 nucleotides. Moreover, we noted that Dbp6 bound with high affinity to a hemi-duplex RNA (ds38/58), resembling a helicase substrate (Supplementary Figure S2A). This hemi-duplex substrate is composed of a 38 bp duplex region with a 20 nucleotide single-stranded 3′ extension. Interestingly, the 38 bp RNA duplex by itself was a poor Dbp6 substrate and the 21-mer oligonucleotide was too short to be bound by Dbp6 (Supplementary Figure S2A), suggesting that the hemi-duplex structure is a better Dbp6 substrate.

As some DEAD-box proteins have the ability to unwind short RNA duplexes, we tested the helicase activity of Dbp6 on substrates consisting of a short 21 nucleotide-long, radiolabeled oligonucleotide base-paired to a longer unlabeled RNA, using the unwinding assay conditions employed to study the Prp43 DEAH-RNA helicase (40,41). We did not detect unwinding activity with the substrate with a long 5′ overhang (Figure 1D, lanes 2–5), and the slight disassembly observed with the substrate with the long 3′ overhang (Figure 1D, lanes 7–10) may not be catalyzed by Dbp6 as it is observed at the start of the incubation and does not increase over time. As we could not detect convincing unwinding activity using this approach, we then used a small hemi-duplex substrate (ds12/30) and a method described to analyze the helicase activity of DEAD-box protein Ded1 (42). This method includes a loading/clamping step of the RNA helicase on the substrate prior to the unwinding step. Dbp6 was pre-incubated for 30 min at 30°C with the hemi-duplex RNA in the presence of ADP, ATP or no nucleotide cofactor. ATP was next added or not to the reactions, which were incubated for another 8 min at 30°C (scheme in Supplementary Figure S2B). We could only observe a very minimal Dbp6 unwinding activity (Supplementary Figure S2B). Finally, we tested whether Dbp6 can unwind a low stability substrate consisting of a radioactive 9-mer RNA oligonucleotide hybridized to the ss30-mer RNA oligonucleotide (Δ*G* = –13.3 kcal/mol). Even with this substrate, we could not detect any helicase activity for Dbp6 (Supplementary Figure S2C). These data show that Dbp6 on its own, under our *in vitro* experimental conditions, either does not unwind RNA substrates, or only very poorly so, suggesting that Dbp6 may not function *in vivo* as a helicase as shown for other DEAD-box proteins (43–46).

### Dbp6 promotes RNA strand annealing to form RNA duplexes

Several DEAD-box proteins have been shown to catalyze RNA strand annealing, in addition, or not, to the duplex unwinding activity (19). This property has been particularly studied in the case of Ded1 and Mss116 proteins (47,48), Rok1 (46) and Laf1/ DDX3X (44) and was speculated to contribute to their function as RNA chaperones (18,49). Considering these examples, we tested whether Dbp6 promotes RNA strand annealing *in vitro*. The ss58-mer RNA was mixed with the complementary radiolabeled ss38-mer RNA in the presence or absence of Dbp6 and, after proteinase K treatment, we monitored hemi-duplex formation over time. As duplex formation can arise spontaneously, we used conditions that do not favor spontaneous annealing, i.e. a low concentration of RNA (Figure 2A, lower panel). We observed that Dbp6 promotes efficient annealing of the two RNA strands (Figure 2A, upper panel and Figure 2B). As controls, incubation of the RNA oligonucleotides with BSA (Figure 2A, lower panel and Figure 2B) or a mock protein purification sample instead of purified Dbp6 (Figure 2B), did not promote duplex formation.

Active annealing relies on two properties of the protein: (i) its ability to bridge two RNA sequences and/or (ii) its ability to prevent spontaneous dissociation of the duplex once formed. To directly demonstrate the ability of Dbp6 to bring together two RNA sequences, we set up the assay described in Figure 2C. We first immobilized a biotinylated RNA oligonucleotide on streptavidin-conjugated beads and then added a second radiolabeled RNA, not complementary to the one immobilized, in the presence or absence of Dbp6. After removing the supernatant, we quantified the amount of radiolabeled RNAs retained on the beads. As the two RNAs are not complementary, the radiolabeled RNAs can only be retained on the streptavidin beads if Dbp6 is able to bridge two RNA strands. Retention of the radiolabeled RNAs added in *trans* was strongly increased over background levels when Dbp6 was added to the reaction (Figure 2D, lanes 1 and 2 and Figure 2E). As additional control, we verified by native gel electrophoresis that the biotinylated and radiolabeled trans RNAs cannot directly hybridize to each other under the conditions used (Supplementary Figure S3A). We conclude from these experiments that Dbp6 is able to bridge two non-complementary RNA sequences, either by binding simultaneously to two RNA molecules or through the interaction of two Dbp6 protomers, each bound to a single RNA molecule. To assess the latter possibility, we tested the ability of Dbp6 to interact with itself *in vivo* by immunoprecipitation experiments with a yeast strain expressing HA-Dbp6 as well as ZZ-tagged Dbp6 (Supplementary Figure S3B). The ZZ tag sequence encodes two tandem repeats of the IgG-binding domain of *S. aureus* protein A. Immunoprecipitation of Dbp6-ZZ using IgG-sepharose, in the presence of RNase, leads to a weak but reproducible co-precipitation of HA-Dbp6 (approximately two-fold enrichment over background, Supplementary Figure S3B, right panel). These results are consistent with the previous detection of a weak *in vivo* Dbp6-Dbp6 interaction in a yeast two hybrid assay (50). Interestingly, the importance of protein-protein interactions for RNA annealing has been demonstrated in the case of the DEAD-box helicases Laf1 of *C. elegans* and its human homolog DDX3X, whose multimerization promotes their RNA annealing activity (44).

In the annealing assay presented in Figure 2A, Dbp6 also likely stabilized the RNA duplex once formed since it displays a substantial affinity for hemi-duplex RNA (Supplementary Figure S2A). To assess the stability of the Dbp6-hemi-duplex interaction, we challenged the complexes formed in EMSAs with increasing amounts of the same unlabeled hemi-duplex RNA (Figure 2F). No release of the radiolabeled hemi-duplex was observed even using a 50-fold excess of the competitor unlabeled hemi-duplex. These observations suggest that Dbp6 binds tightly to RNA hemi-duplexes and may function as an RNA clamp (18,21).

### Dbp6 activities are modulated by ATP or ADP addition *in vitro*

**Figure 3.**
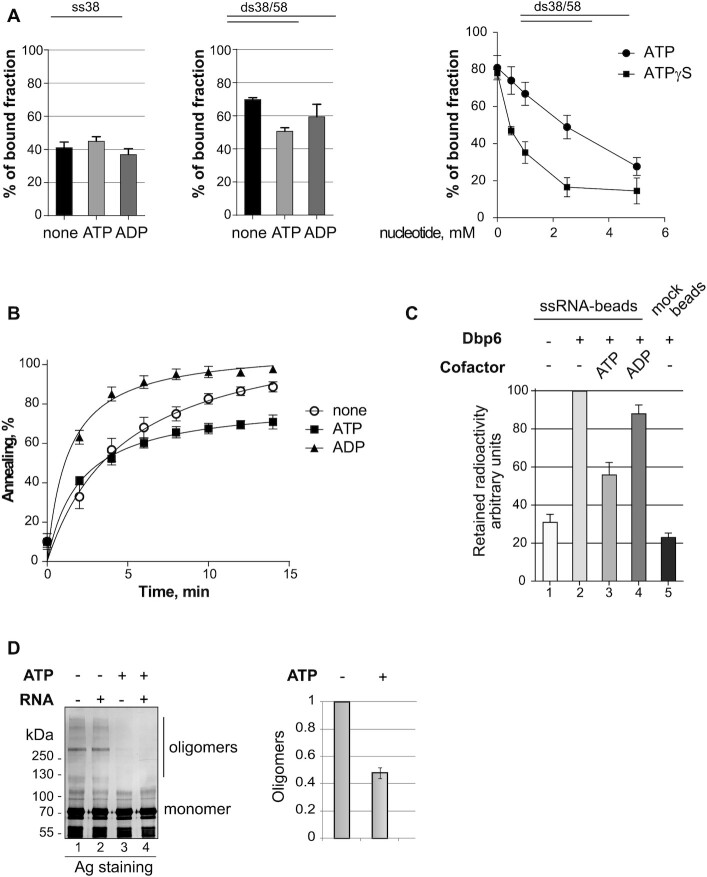
Effect of nucleotides cofactors on Dbp6 activities. (**A**) Effect of ATP, ADP or ATPγS on the RNA binding activity of Dbp6. Binding reactions were performed with a fixed amount of protein (0.5 μM) and RNA (1 nM). Left and central panels: either a ssRNA (ss38-mer), or a hemi-duplex RNA (ss38-mer hybridized to ss58-mer, ds38/58) were respectively used as substrates in the presence or absence of ATP or ADP (2 mM), as indicated. The percentage of bound substrate was plotted for each condition. Right panel: increasing amounts of ATP or ATPγS (0, 0.5, 1, 2.5, 5 mM) were added to the binding reaction with hemi-duplex ds38/58 RNA. The percentage of bound substrate was plotted as a function of nucleotide concentration. (**B**) Effect of ATP or ADP on the annealing activity of Dbp6. Annealing reactions were carried out as in Figure 2B, in time-course experiments with Dbp6 protein (0.25 μM), in the presence of ATP or ADP (5 mM). (**C**) Effect of ATP or ADP on the bridging activity of Dbp6. The reaction followed the scheme described in Figure 2C except that ATP or ADP (4 mM) were added to the reaction, when indicated. The histogram represents the relative quantity of radiolabeled trans-oligonucleotide retained on the streptavidin-beads coated or not with biotinylated oligonucleotide, the amount retained with Dbp6 and no nucleotide being arbitrarily set at 100. (**D**) Effect of ATP on the oligomerization of Dbp6. Purified Dbp6 was incubated with or without ATP (5 mM) in the presence or absence of RNA oligonucleotide, as indicated, before UV cross-linking. On the left panel, the products were separated by native PAGE and visualized by silver staining. On the right panel, the histogram shows the quantity of oligomers in the presence and absence of ATP, the latter being arbitrarily set at 1. Error bars in all cases correspond to SEM for *n* ≥ 3 experiments.

Like other DEAD-box proteins, Dbp6 binds to and hydrolyzes ATP (Figure 1A and B). These events induce conformational changes that could modulate the activity and behavior of the protein (51,52). We evaluated the effects of ATP or ADP addition at saturating concentrations (2–5 mM) on the RNA-binding and annealing activities of Dbp6. We repeated EMSA experiments with ss- and dsRNA oligonucleotides as substrates in the presence of ATP or ADP (Figure 3A). We observed no convincing effect of nucleotide addition on the interaction between Dbp6 and ssRNA (Figure 3A, left panel). In this regard, Dbp6 differs from other DEAD-box proteins (19). However, ATP, and to a lesser extent ADP, reduced the interaction of Dbp6 with the hemi-duplex RNA substrate (Figure 3A, central panel). The negative effect of ATP on hemi-duplex binding was more pronounced with increasing ATP concentrations up to 5 mM (Figure 3A, right panel). We also tested the same range of concentrations of the slowly hydrolyzing ATPγS (Figure 3A, right panel). ATPγS had an even stronger effect on Dbp6 binding to the hemi-duplex than ATP. The different effects of ATP depending on the substrate may reflect different modes of interaction of Dbp6 with ss- or dsRNAs. Indeed, crystal structures of several DEAD-box proteins have consistently shown that ssRNAs bind through a set of conserved residues across both helicase domains, whilst duplex RNA only interacts with the RecA2 domain (19,25,53).

Regarding the annealing activity of Dbp6, we observed that ATP addition had an inhibitory effect (Figure 3B), as shown for Ded1, Mss116 and Rok1 (46–48). However, the inhibition was only partial as the annealing rate was still higher than that observed in the absence of Dbp6 (Figure 2B). In contrast, ADP addition increased the annealing activity of Dbp6. Consistent with this result, a bridging assay also showed that ATP, but not ADP, led to a loss of radioactive RNA retention on the streptavidin beads (Figure 3C, lanes 3 and 4).

Altogether, these results allowed us to conclude that ATP reduces the ability of Dbp6 to bridge RNA sequences. As our results presented in Figure 2D–E and Supplementary Figure S3B suggested that RNA bridging could rely on protomer interactions, we next evaluated whether Dbp6 protomer interactions would be sensitive to ATP. UV cross-linking experiments showed that saturating concentration of ATP (5 mM) reduced Dbp6 oligomerization by ∼2-fold (Figure 3D, lanes 3 and 4 and right panel).

### Mutations in conserved helicase core motifs of dbp6 are detrimental in yeast cells

**Figure 4.**
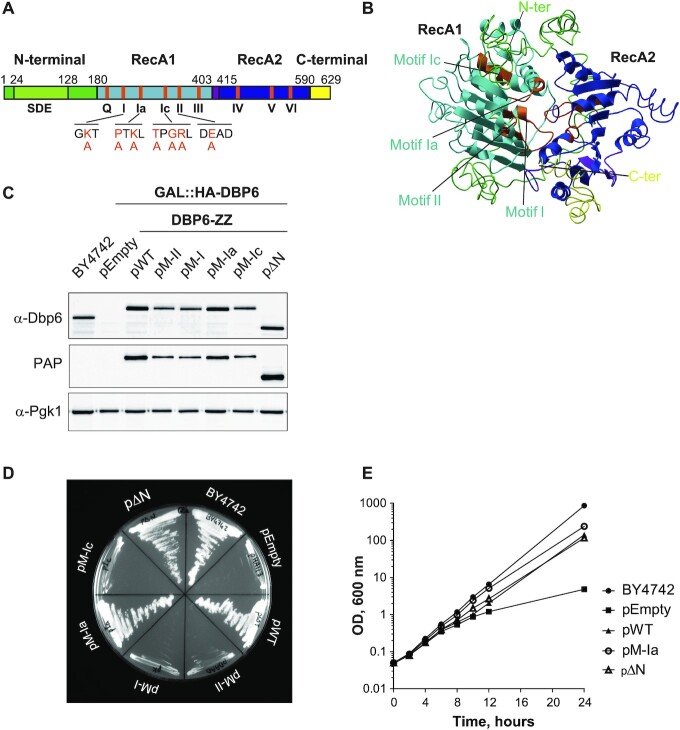
Dbp6 mutants to explore the *in vivo* function of architectural motifs. (**A**) Domain organization of Dbp6. The helicase core domains 1 and 2 are colored in light and dark blue, respectively. The N- and C-terminal extensions are colored in green and yellow, respectively. The sequence spanning residues 24–128 rich in negative amino acids (SDE) present in the N-terminal extension is colored in light green. This SDE sequence was deleted in the ΔN mutant. The locations (in orange) of conserved DEAD-box motifs (6) are shown. Amino acids selected for mutagenesis are highlighted in orange in the conserved sequence below the corresponding motif and the amino acid replacement (alanine) is noted. The domains are drawn to scale. (**B**) Dbp6 structure prediction obtained from different algorithms that use a combination of sequence and structural homologies (Phyre^2^ and i-TASSER servers). The RecA domains are colored as in A). The mutated motifs are shown in orange. (**C**) Immunoblots to assess the expression of Dbp6 mutants. Equivalent amounts of whole cell extracts from GAL::HA-DBP6 cells transformed with the plasmids (pEmpty, pWT, pM-II, pM-I, pM-Ia, pM-Ic, pΔN) or control BY4742 cells grown in glucose-containing medium (15 h) were analyzed by western blot with anti-Dbp6 antibodies or rabbit peroxidase anti-peroxidase (PAP) complex (that detects the ZZ tag). The phosphoglycerate kinase (Pgk1) protein was used as a loading control. (**D**) Growth phenotypes conferred by the Dbp6 mutations. The GAL::HA-DBP6 strains transformed with the plasmids expressing WT Dbp6 (pWT), the different Dbp6 mutants (pM-II, pM-I, pM-Ia, pM-Ic, pΔN), or no protein (pEmpty) were grown on glucose-containing plates. The parental BY4742 strain was also streaked as positive control. (**E**) Growth curves of viable strains expressing Dbp6 mutants from plasmids. Transformed strains and BY4247 control strain were grown in synthetic galactose-containing medium to exponential phase and subsequently shifted to glucose-containing medium. Growth was monitored for 24 h.

To better understand the function of Dbp6 *in vivo*, we analyzed the functional consequences of amino acid changes introduced within key motifs of its helicase core that could differentially impact the biochemical properties of the enzyme. The impact of some amino acid substitutions in Dbp6 have already been reported in the context of a general study of helicases involved in the maturation of pre-60S particles (29). Dbp6 sequence analysis allowed us to identify nine of the conserved motifs described for the RecA-like core of DEAD-box proteins: motifs Q, I, Ia, Ic, II, III, IV, V and VI (Figure 4A). Furthermore, Dbp6 three-dimensional structure prediction (Figure 4B) (54,55) showed a classical DEAD-box protein structure, with the conserved motifs located along the cleft between the two RecA-like modules. Dbp6 also features an N-terminal acidic region, enriched in aspartic acids and glutamic acids (D/E-rich) which is predicted to be intrinsically disordered (56). This region could expose negative charges resembling nucleic acids and could hence modulate the nucleic acid binding capacity of Dbp6. Indeed, according to a large PDB study (57), the D/E-rich repeats were proposed to behave as nucleic acid mimics.

Motifs I (GTGKT) and II (DEAD), also known as Walker A and B motifs, are involved in the binding of nucleoside triphosphates (NTPs) and magnesium, and coordinate NTP hydrolysis (19). They are separated by motifs Ia (PTKL) and Ic (TPGRL), which are involved in nucleic acid binding. The enzymatic roles of specific residues within these motifs were deduced from mutagenesis and biochemical analyses (18). On this basis, we chose to substitute by alanines (A) the lysine (K) of motif I (M-I mutant), the glutamic acid (E) of motif II (M-II mutant), the proline (P) and lysine (K) of motif Ia (M-Ia mutant), and the tyrosine (T), glycine (G) and arginine (R) residues of motif Ic (M-Ic mutant), as illustrated in Figure 4A. In addition, we generated a version of Dbp6 lacking the N-terminal D/E-rich sequence spanning residues 24–128 (ΔN mutant). The localization of the motifs affected by the mutations is indicated on the tertiary structure prediction (Figure 4B).

As Dbp6 is an essential protein, mutations affecting functionally important domains could have severe consequences on cell viability. To express and study *DBP6* mutants, we used a yeast strain featuring a conditional expression system in which expression of the endogenous *DBP6* open reading frame is under the control of a galactose-inducible promoter and tagged with HA (GAL::HA-DBP6). We then transformed this strain with expression plasmids bearing wild-type or mutated versions of *DBP6* (as described above), fused at its 3′ end to the ZZ tag sequence. In the resulting strains, endogenous Dbp6 is expressed in a galactose-containing medium, and its expression is turned off once cells are grown in the presence of glucose, where only the plasmid-borne genes are expressed (Supplementary Figure S4A). This expression system allowed us to follow the effects of Dbp6 amino acid substitutions on cell viability and ribosome biogenesis.

To assess the expression levels of wild-type and mutant Dbp6 proteins after transfer to the glucose-containing medium, we first defined the culture conditions by shifting for different times the GAL::HA-DBP6 strain into glucose medium followed by western blot analyses using polyclonal antibodies to detect Dbp6. We observed that HA-Dbp6 was over-expressed in the presence of galactose compared to its endogenous levels in the BY4742 control strain, and became undetectable after 3 hours of growth in the glucose-containing medium (Supplementary Figure S4A). We then performed western analyses with extracts from GAL::HA-DBP6 cells transformed with plasmids encoding wild-type or mutant versions of Dbp6-ZZ (Figure 4C) and depleted of chromosome-encoded HA-Dbp6 by growth in glucose-containing medium. We observed that all mutant Dbp6 proteins were expressed and that the ΔN mutant accumulated to higher levels than all the others.

The growth phenotypes of the different strains were evaluated on solid media or in liquid cultures. Similar to cells transformed with the empty vector (pEmpty), cells containing the plasmid-encoded M-II, M-I and M-Ic mutations, failed to grow on glucose-containing plates (Figure 4D), as reported for Dbp6-depleted cells (35) indicating that the amino acid substitutions introduced in motifs I, Ic and II impair viability. Cells carrying plasmids pM-Ia and pΔN were viable on glucose-containing medium, but their generation time in liquid cultures (118 min and 132 min, respectively) were increased compared to the BY4742 wild-type strain (102 min, Figure 4E). However, cells transformed with the plasmid carrying wild-type *DBP6* showed a similar growth defect (130 min), potentially due to the presence of the ZZ tag. It therefore remains unclear whether the slight growth defects of the M-Ia and ΔN mutants are due to the ZZ tag, direct effect of the mutations, or a combination of these features.

Finally, we tested whether increased levels of the DEAD motif mutant (M-II) in cells also expressing wild-type Dbp6 is detrimental to cell growth. To do so, we transformed a wild-type strain with vectors encoding GFP-tagged Dbp6 (Dbp6-GFP) or Dbp6 DAAD (M-II-GFP) under the control of an oestradiol-regulated promoter, or the corresponding empty vector as control. Addition of 10 mM oestradiol led to the same levels of GFP-tagged Dbp6 as those of endogenous Dbp6, while increasing the concentration of oestradiol to 100 mM resulted in a clear overexpression of GFP-tagged Dbp6 (Supplementary Figure S5A). The growth of the transformed strains in the presence of 10 or 100 mM oestradiol was then assessed by spot assays (Supplementary Figure S5B). At 100 mM oestradiol, growth of cells expressing the M-II-GFP mutant was clearly affected, while that of cells expressing wild-type Dbp6-GFP was not. Our results are consistent with those obtained by Bernstein and colleagues (29) and we conclude that the Dbp6 M-II mutant affects cell growth in a dominant-negative manner.

### Dbp6 mutations impair early pre-rRNA processing

**Figure 5.**
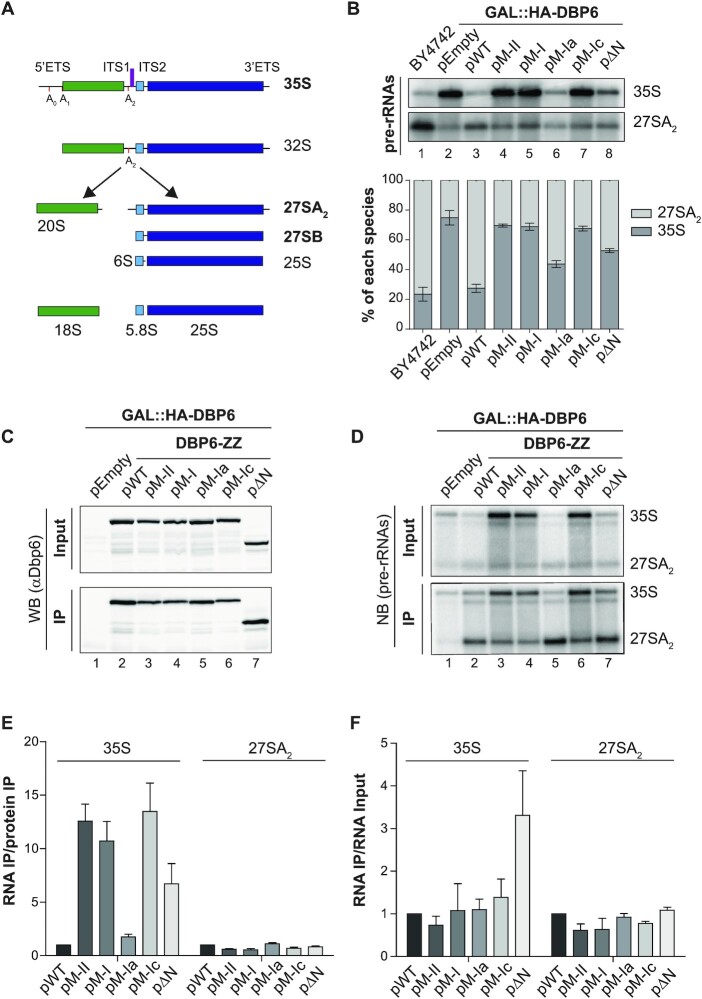
Dbp6 mutants affect processing of early large subunit rRNA precursors. (**A**) Simplified scheme of the yeast pre-rRNA processing pathway. The 35S primary transcript contains the sequences of the mature 18S (in green), 5.8S (in light blue) and 25S rRNAs (in dark blue) separated by two internal transcribed spacers (ITS1 and ITS2), and flanked by two external transcribed spacers (5′ETS and 3′ETS). The location of the early cleavage sites (A_0_, A_1_ and A_2_) are indicated by red vertical bars. The probe used in northern blots for detection of pre-rRNAs is represented as a purple bar in the ITS1. Names of the pre-rRNAs that are relevant to this study are written in bold. (**B**) Analysis of pre-rRNA processing in yeast cells expressing WT or mutant Dbp6 from a plasmid. Equal amounts of total RNAs (3 μg) extracted from glucose-containing cultures were analysed for the steady-state levels of pre-rRNAs by northern blot (with the 23S.1 probe, Supplementary Table S3). ‘BY4742’ corresponds to RNAs extracted from the parental strain devoid of any plasmid. Relative levels of 35S and 27SA_2_ pre-rRNAs are shown in the histogram below the northern blot. (C–F) Immunoprecipitation experiments (IPs) to analyse the association of Dbp6 mutants with pre-ribosomal particles. IPs were performed with IgG-sepharose and extracts from GAL::HA-DBP6 cells transformed with the indicated plasmids and grown in glucose-containing medium. Total proteins and RNAs were extracted from Input and precipitated (IP) samples and analysed by western (**C**) and northern (**D**) blotting, respectively. (**C**) Western blot analysis: levels of WT and mutant Dbp6-ZZ in Input and IP samples were determined using anti-Dbp6 antibodies. (D) Northern analysis: early pre-rRNAs (35S and 27SA_2_) from Input or IP samples were specifically identified with the 23S.1 probe (Supplementary Table S3). (E, F) The pre-rRNA levels in IP samples normalized to precipitated protein bait levels are given in the histogram (**E**) below the western blot, while the ratios of the pre-rRNA levels in IP samples over those in Input samples are presented in the histogram (**F**) below the northern blot. The ratios were standardized to those obtained for the WT protein, arbitrarily set at 1. Error bars in all cases correspond to standard deviation (SD) for *n* ≥ 3 experiments.

Dbp6 is required for the conversion of 90S pre-ribosomal particles into early pre-60S particles (29,35). The absence of Dbp6 leads to the accumulation of the 35S pre-rRNA contained in the 90S particle, and the depletion of the downstream processing product, the 27SA_2_ pre-rRNA (Figure 5A) (29,35). We confirmed these data in preliminary experiments where we shifted the GAL::HA-DBP6 strain, or the wild-type BY4742 strain as a control, from a galactose- to a glucose-containing medium and analysed pre-rRNA processing after increasing times of growth on glucose (Supplementary Figure S4B). Dbp6 depletion led to a strong accumulation of the 35S pre-rRNA and a depletion of the 27SA_2_ intermediate starting from 6 h after transfer to the glucose-containing medium. The same effects were observed with the lethal mutants M-II, M-I and M-Ic (Figure 5B, lanes 4, 5 and 7), and the relative quantification of the 35S and 27SA_2_ pre-rRNAs in the mutants (Figure 5B, lower panel) revealed ratios comparable to that observed in the absence of Dbp6 (pEmpty, lane 2). The M-Ia mutation and the ΔN truncation induced similar, but attenuated, effects on pre-rRNA profiles (Figure 5B, lanes 6 and 8). In conclusion, the amino acid substitutions and truncation introduced in Dbp6 affect the conversion of the 35S pre-rRNA into 27SA_2_ to extents proportional to the growth defects.

The amino acid substitutions in Dbp6 could interfere with pre-rRNA processing either because the mutant proteins are not incorporated into pre-ribosomes or because they cannot fulfil their function once recruited. To determine whether the mutant/truncated Dbp6 proteins were present within pre-ribosomal particles, immunoprecipitation (IP) experiments were performed using extracts from yeast cells expressing wild-type or mutant ZZ-tagged Dbp6 proteins. The precipitation of the ZZ-tagged proteins was checked by western blot (Figure 5C) and the co-precipitation efficiency of pre-rRNAs was analysed by northern blot (Figure 5D). Quantification of the ratios of the immunoprecipitated RNAs over the immunoprecipitated proteins (Figure 5E) revealed that the 35S pre-rRNA was co-precipitated more efficiently with all the mutant proteins (the M-Ia mutant to a lesser extent) compared to the wild-type situation. Although expression of the Dbp6 mutants causes 35S pre-rRNA accumulation (Figure 5B), this result was a first indication that the mutant Dbp6 proteins are efficiently incorporated into 90S particles. Comparatively, the 27SA_2_ pre-rRNA was co-immunoprecipitated with lower efficiencies with mutant Dbp6 proteins. Comparing the immunoprecipitation efficiencies of the 35S and 27SA_2_ pre-rRNAs relatively to the input samples showed that wild-type Dbp6 is associated with the 35S and 27SA_2_ pre-rRNAs and that these intermediates are also co-immunoprecipitated with most Dbp6 mutants with similar efficiencies (Figure 5F). The only exception is the ΔN truncated mutant, which showed a 3-fold increased IP efficiency for the 35S pre-rRNA. This could suggest that Dbp6 lacking the N-terminal region associates more stably with the 35S pre-rRNA.

To confirm this result using other experimental means, we performed Ribo Mega-SEC experiments (58) to fractionate the (pre-)ribosomal particles by size-exclusion chromatography and analyzed the elution profiles of wild-type or mutant versions of Dbp6 using western blot analyses (Supplementary Figure S6). The different fractions contained particles ranging from polysomes, 80S monosomes, free ribosomal subunits and uncharacterized small complexes. We observed that the overall elution profiles obtained with extracts from cells expressing the mutant versions of Dbp6 were different from that obtained with the wild-type cells. The peak heights on the elution absorbance profiles (upper panel) were decreased, likely reflecting a defect in ribosome synthesis and translation. Among the 13 collected fractions (fraction 16 to fraction 28), the wild-type Dbp6 protein was present in the fraction containing the (pre-)40S and (pre-)60S subunits (Supplementary Figure S6, fractions 23 and 24) as well as in fractions containing smaller complexes, while the M-II, M-I and ΔN mutants were also present in fractions containing larger particles, most likely early 90S particles (Supplementary Figure S6, fractions 20–22). Together with the co-immunoprecipitation experiments described above, these observations suggest that the mutant Dbp6 proteins are present in the 90S particles that accumulate and in the residual pre-60S particles.

### Dbp6 mutants display abnormal biochemical properties

The co-immunoprecipitation and Ribo Mega-SEC experiments suggest that the mutant Dbp6 proteins are integrated into pre-ribosomal particles, and that the pre-rRNA processing inhibition observed upon their expression is likely caused by defects in the activities of the mutant proteins. To explore the nature of these defects, we expressed and purified recombinant versions of each Dbp6 mutant following the same procedure as for the wild-type protein. Upon size exclusion chromatography, the majority of proteins eluted in two major peaks, corresponding to oligomeric and mono-/dimeric species according to the molecular weight standards used to calibrate the column (see e.g. M-II and ΔN mutants; Supplementary Figure S7A, B). Depending on the protein, the calculated ratio between these two peaks differed, with a large oligomer prevalence for all mutants (M-II, M-I, M-Ia and M-Ic), except for the ΔN mutant (Supplementary Figure S7B, C). This observation suggests that the mutations introduced in domains I and II of Dbp6 favour oligomerization. For the M-Ia and M-Ic mutants, it was not possible to obtain a mono-/dimeric fraction that would allow activity assays to be performed. The fact that all mutants, except the ΔN, were reproducibly more inclined to form high-order oligomers suggests that oligomerization reflects intrinsic states of the mutant proteins rather than purification artefacts.

**Figure 6.**
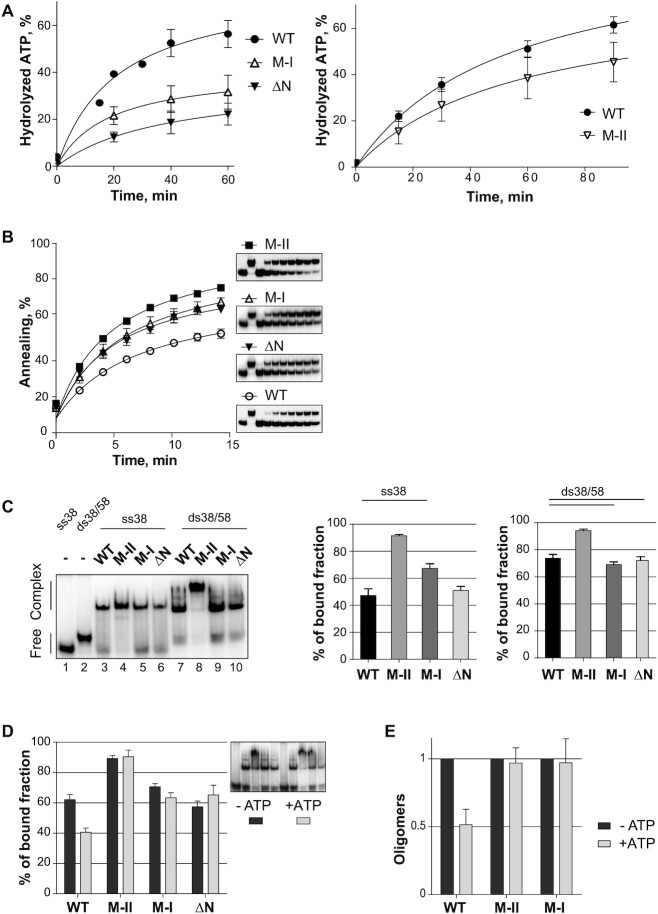
Biochemical analysis of the Dbp6 mutants. (**A**) ATPase activity. The assays were performed as described in Figure 1B, except for the concentration of protein, which was 0.5 μM for the left graph, and 0.3 μM for the right graph. The M-II protein concentration was lowered to remain in the same reaction volume because the yield of the purified M-II protein was constantly lower than that of the other mutants. Both assays were performed in the presence of RNA oligonucleotide (2 μM). (**B**) Time course experiments to assess annealing activity. The assays were performed as described in Figure 2B, except that less proteins were used (0.025 μM). Insets: representative PAGE analysis of annealing reaction products performed as described in Figure 2A. (**C**) RNA binding capacity. EMSAs using either ss-oligonucleotide (ss38-mer, lanes 3–6) or hemi-duplex oligonucleotide (ds38/58, lanes 7–10) as substrates were carried out with the WT and mutant Dbp6 proteins. For binding to ss38-mer, protein concentration was 0.5 μM, except for M-II (0.25 μM). For binding on ds38/58, protein concentration was 0.25 μM. Free oligonucleotides, ss38-mer (lane 1) and ds38/58 (lane 2) were loaded as migration controls. Histograms on the right present the average of the percentage of the bound fraction. (**D**) Effect of ATP on RNA binding. The same EMSAs on hemi-duplex substrate as in C) were repeated in the presence or absence of ATP (5 mM). The histogram presents the average of the percentage of the bound fraction. A representative EMSA is shown in the inset. (**E**) Effect of ATP on the oligomerization capacity of the mutants. UV cross-linking experiments were performed as described in Figure 3D except that no RNA was included. The histogram shows the quantity of oligomers obtained with the indicated proteins in the presence and absence of ATP, the latter being arbitrarily set at 1. Error bars in all cases correspond to SEM for *n* ≥ 3 experiments.

ATP binding and/or hydrolysis induce conformational changes within the RNA helicase core that regulate activity (52,59). We evaluated the capacity of the mutant proteins to hydrolyse ATP. The ATPase assays showed a clearly reduced activity for the M-I mutant compared to the wild-type protein (*V*_max_ = 41.35 ± 13.93 μM/min and *V*_max_ = 77.69 ± 11.82 μM/min respectively, Figure 6A), and the activity of the ΔN mutant was even further affected (*V*_max_ = 36.43 ± 19.12 μM/min). We were expecting the M-I mutant to display only background ATPase activity. The fact that the ATPase activity obtained was higher than that seen with the ‘pET + RNA’ control (Figure 1B) suggests that trace amounts of an *E. coli* ATPase may co-purify with monomeric/dimeric Dbp6 and its mutants (see also Discussion). For the ATPase assay with the M-II mutant, we had to change the reaction conditions because the yield of purified protein was constantly lower than for the other mutants. We had to use less M-II protein (0.3 μM versus 0.5 μM), and compensated with a longer time course. In these conditions, the activity of the M-II mutant was also affected but to a lesser extent than the other mutants (Figure 6A, right panel). We conclude that all the tested Dbp6 mutants show a reduced ATPase activity, either due to their inability to bind ATP or to hydrolyse it.

We next tested the effect of the mutations on Dbp6 RNA annealing activity as described above (Figure 2B). All the mutant proteins showed a more efficient annealing activity compared to WT Dbp6 (Figure 6B). Note that the protein concentrations were lowered compared to Figure 2 to accommodate the increased activity of the mutants. We observed in particular at the end of the time-course experiment a >50% increase in duplex formation with the M-II mutant compared to the WT protein. This enhanced annealing activity of the M-II mutant, and to a lesser extent of the M-I mutant, might be related to their elevated propensity to self-associate (Supplementary Figure S7). It could also result from a higher affinity for RNA and/or a more stable interaction with hemi-duplex structures. To verify this hypothesis, we monitored the RNA-binding properties of the mutants using both single-stranded and hemi-duplex RNA oligonucleotides (Figure 6C). The M-II mutant clearly showed a stronger binding to both RNA substrates. The increased affinity for the ssRNA was such that we used half the amount of M-II protein compared to WT to remain in the same range of binding efficiency. Likewise, but to a lesser extent, the Dbp6 M-I mutation also increased the binding capacity of the protein on ssRNA but not on the hemi-duplex. The ΔN protein behaved like the wild-type protein in binding to both ss- and hemi-duplex RNAs. This was a somewhat unexpected result as it is possible that the N-terminal D/E-rich domain might function as a ‘nucleic acid mimic’ negatively regulating the RNA-binding properties of Dbp6.

We have shown above that ATP decreases the annealing activity of wild-type Dbp6 (Figure 3B), which was correlated with a reduction in the hemi-duplex RNA binding capacity and a reduction of the oligomerization of the protein. Considering these data, we have refined the biochemical study of the mutants by evaluating the influence of ATP on the RNA binding activity and oligomerization capacity of the mutant Dbp6 proteins. Our results indicated that the presence of ATP did not change their binding to hemi-duplex RNA (Figure 6D), contrary to the wild-type protein. Likewise, ATP did not reduce the oligomerization of the mutant proteins (Figure 6E), contrary to the observation with the wild-type protein.

### Identification of the dbp6 binding sites on pre-rRNAs and snoRNAs

As Dbp6 is incorporated into early pre-ribosomal particles (Figure 5), the protein potentially contacts directly pre-rRNA sequences, snoRNAs and/or pre-rRNA/snoRNA hybrids. To identify cross-linking sites of wild-type Dbp6 on cellular RNAs, the cross-linking and analysis of cDNA (CRAC) method (38) that has been successfully used for other RNA helicases (10–11,14,16,60–62) was employed. A plasmid encoding a His_6_-TEV-Protein A (HTP)-tagged version of Dbp6 was introduced into the GAL::HA-DBP6 strain. After *in vivo* UV-cross-linking, cell extracts were prepared and processed according to the CRAC protocol. Briefly, early pre-ribosomal particles containing Dbp6 were isolated under native conditions and after partial RNase digestion, Dbp6-containing complexes were purified under denaturing conditions. Adaptor sequences were ligated at the 5′ and 3′ ends of the Dbp6-associated RNA fragments, which were then copied to cDNAs by reverse transcription. The resulting cDNA library was amplified by PCR and deep-sequenced using Illumina sequencing.

Initial analysis of the distribution of the sequences recovered with Dbp6 showed that most of them (70%) derive from rRNA sequences (Supplementary Figure S8A), which were also abundant (over 80%) in the control experiment performed with cells devoid of tagged Dbp6 (BY4742 strain). The large majority of these sequences likely correspond to contaminating mature rRNA sequences recurrently observed in CRAC experiments.

**Figure 7.**
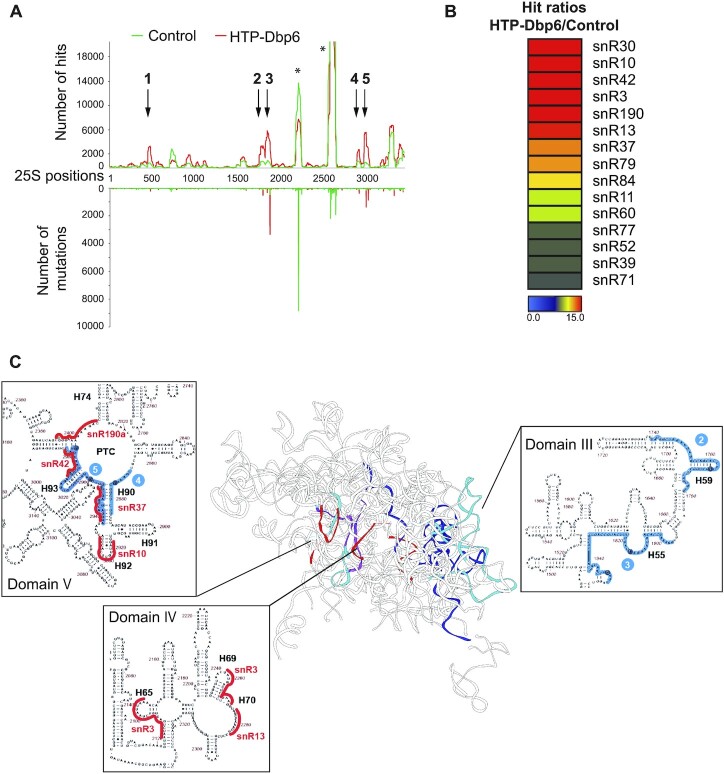
Interactions of Dbp6 with the 25S rRNA and snoRNAs determined by CRAC. (**A**) Distribution of unique sequence reads of Dbp6 CRAC experiment on the 25S rRNA. The number of reads (upper graph) and the number of mutations/deletions (lower graph), mapping to each nucleotide of the rDNA sequence coding for the 25S rRNA, are plotted for the control (green) and HTP-Dbp6 (red) CRAC experiments (Plots for the entire RDN37 rDNA region are given in Supplementary Figure S8). The positions of mutations/deletions commonly correspond to precise binding sites. The major Dbp6 cross-linked sites (with hit values at least four-fold above the untagged control sample) are numbered (1 to^5^). Asterisks indicate common contaminating peaks. (**B**) Heat map generated from the fold enrichments of snoRNAs in the HTP-Dbp6 CRAC data set versus control sample data set using Genesis (from Graz University of Technology). The grading is such that only the snoRNAs with a fold enrichment of 2.65 or more, and 700 or more reads per million are considered. The detailed numbers of hits are given in Supplementary Figure S9A. (**C**) The HTP-Dbp6 cross-linked sites (in blue) and the target sites of the major Dbp6 bound snoRNAs (in red) are positioned on the rRNA 3D structure of the state D nucleolar pre-60S particle (PDB code: 6EM5) (65). The insets correspond to partial maps of the 25S 2D structure, with the localizations of these Dbp6 cross-linking sites and snoRNA binding sites highlighted. The complete annotated 25S 2D map is given in Supplementary Figure S8.

Alignment of the sequencing reads obtained with Dbp6 or in the control experiment (untagged BY4742 strain) with the sequence of the full-length 35S pre-rRNA transcript revealed that the most obvious differences in peak intensities lie in the 25S rRNA sequence (Supplementary Figure S8B), consistent with the function of Dbp6 in pre-60S particle maturation. Precise comparison between the 25S rRNA read profiles allowed to identify five peaks (annotated from 1 to 5) specific to the Dbp6 CRAC experiment (Figure 7A). Their biological significance was supported by the presence of point mutations or deletions, which likely correspond to the physical contact points of the protein on the RNAs (Figure 7A). These five peaks mapped in three discrete regions of the 25S rRNA, helices H90/H93 in domain V, helices H51/H54/H55/H59 in domain III (Supplementary Figure S8C and Figure 7C) and the third one in domain I. Interestingly, two of these regions (I and V) where Dbp6 cross-linking sites are found were also reported as interaction sites for Npa1 (36), its associated snoRNA chaperone snR190 (12) and for the Dbp7 helicase involved in remodeling events in the PTC region (16), supporting a functional relationship between these different factors involved in early pre-60S particle assembly.

Interestingly, the CRAC experiments further revealed a strong enrichment of several snoRNA sequences among the RNA fragments cross-linked to Dbp6, compared to the BY4742 control (Supplementary Figures S8A and 7B). This observation suggests that the function of Dbp6 could be related to regulating snoRNA dynamics on pre-ribosomes, as reported for other RNA helicases (10–11,13–16,62–64). The list of these snoRNAs is presented in Figure 7B as a heat diagram reflecting the enrichment of the snoRNA sequencing reads in the Dbp6 CRAC experiment compared to the control. The numbers are detailed in Supplementary Figure S9A and the profiles of the Dbp6 CRAC reads on the sequence of the top list snoRNAs is shown in Supplementary Figure S9B. The base-pairing sites of these snoRNAs on the 25S rRNA are shown in Figure 7C and Supplementary Figure S8C. Two interesting observations emerged from the analysis of these snoRNAs. First, the base-pairing sites of the different snoRNAs lie adjacent to each other in two areas of the 25S rRNA: domain V (snR10, snR37, snR42 and snR190a) and domain IV (snR3, snR13). Second, the snoRNAs hybridizing to domain V (snR10, snR37, snR42 and snR190) are clustered in the vicinity of the 25S rRNA fragments cross-linked to Dbp6 (Figure 7C).

It is not as yet possible to determine where Dbp6 pre-rRNA binding sites and Dbp6-interacting snoRNAs are positioned in the first pre-60S particle, as no high resolution structure of such a particle is available. As a substitute, we examined where these Dbp6 binding sites and snoRNAs are positioned on the structure of the so-called ‘state D’ nucleolar pre-60S particle (PDB code: 6EM5, Figure 7C) (65). In this structure, the distances between Dbp6 cross-linking sites in domain V and domain III vary between 76 and 123 Å. As the region of domain I that contains the Dbp6 cross-linking site at the nucleotide N467 is missing from the structure, we performed measurements using nucleotide 495 instead. We measured a distance of 105 Å between nucleotide 495 of domain I and the cross-linking site in helix H59 of domain III (nucleotide U1765). Considering that the predicted Dbp6 structure (solved by alpha-fold) spans 103 Å, a dimer of Dbp6 may contact simultaneously several cross-linking sites.

Taken together, our results suggest that Dbp6 functions in the neighborhood of the PTC region during the early stages of pre-60S particle formation.

## DISCUSSION

To be competent for protein synthesis, ribosomes need to be correctly assembled. The last decade has brought large progress in the understanding of the production of ribosomes with the resolution of cryo-EM structures of pre-ribosomal particles (3,65–68). However, structural information on very early pre-60S particles is still sparse (31). Their probable high flexibility likely makes structural analyses particularly challenging. Very early pre-60S particles contain numerous snoRNPs and AFs, including several DEAD-box proteins (31,69). One of these is Dbp6, required for normal steady-state accumulation of the first pre-60S particle (35,36). We aimed at deciphering the mechanism of action of this putative helicase to shed light on how it may contribute to the critical first steps of large ribosomal subunit biogenesis. To decipher the mode of action of Dbp6, we undertook an extensive *in vitro* study of Dbp6 biochemical activities and performed *in vivo* analyses of the effects of Dbp6 mutations. We demonstrate that Dbp6 is endowed with ATPase, RNA strand annealing and clamping activities that are probably important for its function. Indeed, our data also show that the core motifs implicated in ATP binding and hydrolysis are essential for Dbp6 function in ribosome biogenesis. Our identification of Dbp6 RNA binding sites by CRAC leads us to propose that the biochemical activities of Dbp6 contribute to the early folding of the 25S rRNA PTC region and/or the regulation of snoRNA/PTC interactions.

DEAD-box proteins can remodel RNA and RNP complexes and assist RNA folding. Indeed, some members of the helicase family facilitate RNA folding by conferring stability to thermodynamically unstable conformations and/or by resolving misfolded intermediates. The ability of RNAs to adopt precise three-dimensional structures is critical for biological processes, and is highly relevant during ribosome biogenesis. It has been proposed that RNAs whose functions depend on multiple conformations may rely on DEAD-box proteins acting as clamps to increase the populations of necessary but less stable conformations, thereby increasing their overall efficiencies (70). During ribosome biogenesis, the target sites of DEAD-box proteins would presumably be short duplexes within higher-order RNA structures. Dbp6 contains the canonical protein motifs conferring to RNA helicases their inherent abilities to bind to RNA and ATP and to hydrolyse ATP. Our biochemical data show that indeed, Dbp6 binds to RNA with a preference for single-stranded and hemi-duplex molecules, and behaves as an RNA-dependent ATPase *in vitro*. In our *in vitro* assay conditions, we were unable to detect a substantial unwinding activity associated with purified Dbp6, even using substrates with very short duplex regions similar to other DEAD-box ATPases, such as the yeast protein Rok1 (46) or the DDX3 ortholog in *C. elegans*, Laf1 (44). This result suggests that Dbp6 may not function as a RNA helicase *in vitro* and *in vivo*, or alternatively that co-factors are required to confer helicase activity to Dbp6. Several DEAD-box helicases have been shown to unwind RNA duplexes efficiently in partnership with co-factors (28). The best-characterized example is the eukaryotic translation factor eIF4A with its cofactors eIF4B or eIF4G (71–74). Similarly, as Dbp6 belongs to the Npa1 complex (36), its protein partners in the complex would represent obvious candidates to provide a potential stimulation of Dbp6 unwinding activity. However, Dbp6 may act as a RNA annealase rather than a helicase *in vivo* since our data show that Dbp6 is endowed with a RNA strand annealing activity *in vitro*. Such a function would not be unprecedented, as the Rok1 helicase was reported to carry out an annealing reaction that specifically promotes formation of a duplex between ITS1 and helix H44 *in vivo*, in the presence of Rrp5 (46,75). Dbp6 facilitates the annealing of complementary RNA strands under unfavourable base-pairing conditions (i.e. diluted RNAs) and has also the ability to bridge non-complementary RNA strands. Our data further suggest that the RNA annealing activity of Dbp6 relies on at least three properties of the enzyme: its single-stranded RNA binding capacity, its oligomerization, and its ability to bind tightly to hemi-duplex RNAs. Oligomerization and clamping have already been described for DEAD-box proteins (19,42,76), but their contribution to RNA annealing has only been suggested once (44). Based on our biochemical data, this annealing activity may depend either on the ability of a single Dbp6 protomer to bind simultaneously to two RNA strands, or on the oligomerization of Dbp6 protomers each bound to a ssRNA strand. Following annealing, Dbp6 could function as a clamp to stabilize the RNA duplex. Given that ATP negatively regulates the RNA binding and oligomerization of Dbp6, it is possible that ATP binding and/or hydrolysis may allow Dbp6 to dissociate from the RNA duplex when appropriate during the pre-ribosome maturation pathway.

Interestingly, our biochemical data show that mutations in motifs I and II of Dbp6 do not fully abrogate the ATPase activity of the enzyme in contrast to mutations in the same motifs of other DEAD-box proteins that have often been described as leading to a complete loss of ATPase activity. Technical issues could account for this discrepancy, in particular regarding recombinant protein purification. Most studies used one-step affinity purification procedures while we used a two-step purification scheme consisting of a nickel affinity purification followed by a size-exclusion chromatography. According to our purification results, the M-I and M-II mutants were predominantly purified as oligomers (Supplementary Figure S7), whereas the wild-type protein eluted mostly as a mono-dimeric form. Our ATPase assays using the different fractions of the size-exclusion chromatography column for the wild-type Dbp6 protein showed that the oligomeric forms are less active than the mono-dimeric forms. We thus conclude that oligomerized proteins are not in a favourable conformation for ATP hydrolysis. The detection of some low levels of ATPase activity with the mono-dimeric forms of the M-I and M-II mutants, but not with the oligomeric fractions, may indicate that the M-I and M-II mutant Dbp6 proteins retain some ATPase activity, which could have been overlooked if only the oligomeric fractions had been used. When using only a one step purification scheme, the mutant protein preparation might mostly contain oligomers and appears as catalytically dead. However, we cannot rule out the possibility that trace amounts of an *E. coli* ATPase may co-purify with monomeric/dimeric wild-type Dbp6 and its mutants.

Our phenotypic analysis in yeast showed that Dbp6 mutations in the conserved motifs affect the transition between 90S particles containing the 35S pre-rRNA and early pre-60S particles containing the 27SA_2_ intermediate. These defects are most likely related to the changes in the biochemical activities of the mutant Dbp6 proteins. Mutations in motifs I and II (M-I and M-II), which prevent viability *in vivo*, yield Dbp6 proteins showing increased RNA binding and annealing activities *in vitro*, and which are no longer responsive to ATP. These modified biochemical features could either generate aberrant RNA duplexes in the pre-ribosomal particles or prevent the timely dissociation of RNA duplexes during the maturation process resulting in longer-lived base-pairings. Both of these defects could impair normal pre-rRNA maturation and account, directly or indirectly, for the lethal phenotype.

To identify the substrates of Dbp6 activities in pre-ribosomal particles, we performed CRAC experiments. Some of the Dbp6 pre-rRNA binding sites are localized in the vicinity of those of Npa1 in domains I and V (36), while others are more distant. Dbp6 also interacts with a subset of snoRNAs, some of which are also bound by Npa1 (36), and whose pre-rRNA target sequences are localized in the vicinity of the PTC region in domain V. Dbp6 interacts with another set of snoRNAs hybridizing to a region of domain IV of the 25S rRNA that is positioned near domain V in the 3D structure of nucleolar pre-60S particles in the so-called state D (65). This physical proximity of Dbp6 and Npa1 pre-rRNA binding sites, combined with their numerous genetic and biochemical interactions, strongly suggest that Dbp6 and its partners in the Npa1 complex cooperate in initiating the structuring of domains I, V and VI during formation of the first pre-60S particle. The first event in the production of the large subunit is the formation and the stabilization of domain I, involving base-pairing between the 5′end of 25S rRNA and 5.8S rRNA (33). The next steps appear to be the gradual clustering with domain I of domain II, then VI followed by V, and their stabilization, as suggested by cryo-EM structures of the likely most upstream maturation products of the first pre-60S particle (31,65,67–68). By establishing a physical bridge between the 5′ (domain I) and 3′ ends (domains V and VI) of the 25S rRNA, the Npa1 complex and its associated snoRNAs in the first pre-60S particle might promote the circularization of the 25S rRNA and in so doing pave the way for the future clustering and stabilization of domains I, II, VI and V. Npa1 and Npa2 are very large proteins predicted to adopt extended alpha-solenoid structures potentially able to contact several distant pre-rRNA regions and thus well suited to perform a structural as well as bridging function. Moreover, our results suggest that Dbp6 may contribute to regulating snoRNA/pre-rRNA interactions and/or structuring the PTC region at the interface between domain IV and domain V by providing RNA strand annealing and clamping activities.

## DATA AVAILABILITY

The CRAC datasets and their analyses for Dbp6-HTP and the wild-type yeast control are deposited in Gene Expression Omnibus (GEO) database [http://www.ncbi.nlm.nih.gov/geo/] under the accession code GSE200692. Other data supporting the findings of this study are available from the corresponding authors upon reasonable request.

## Supplementary Material

gkac1196_Supplemental_File

## Notes

Present address: Ali Khreiss, Department of Molecular Biology, University Medical Center Göttingen, 37073 Göttingen, Germany.

## References

[1] Henras A.K. , Plisson-Chastang C., O’Donohue M.F., Chakraborty A., Gleizes P.E. An overview of pre-ribosomal RNA processing in eukaryotes. Wiley Interdiscip. Rev. RNA. 2015; 6:225–242. 25346433 10.1002/wrna.1269 PMC4361047

[2] Woolford J.L. Jr , Baserga S.J Ribosome biogenesis in the yeast *Saccharomyces cerevisiae*. Genetics. 2013; 195:643–681. 24190922 10.1534/genetics.113.153197 PMC3813855

[3] Klinge S. , Woolford J.L. Jr Ribosome assembly coming into focus. Nat. Rev. Mol. Cell Biol. 2019; 20:116–131. 30467428 10.1038/s41580-018-0078-y PMC7725133

[4] Kressler D. , Hurt E., Bergler H., Bassler J. The power of AAA-ATPases on the road of pre-60S ribosome maturation–molecular machines that strip pre-ribosomal particles. Biochim. Biophys. Acta. 2012; 1823:92–100. 21763358 10.1016/j.bbamcr.2011.06.017 PMC3264779

[5] Sloan K.E. , Warda A.S., Sharma S., Entian K.D., Lafontaine D.L.J., Bohnsack M.T. Tuning the ribosome: the influence of rRNA modification on eukaryotic ribosome biogenesis and function. RNA Biol. 2017; 14:1138–1152. 27911188 10.1080/15476286.2016.1259781 PMC5699541

[6] Fairman-Williams M.E. , Guenther U.-P., Jankowsky E. SF1 and SF2 helicases: family matters. Curr. Opin. Struct. Biol. 2010; 20:313–324. 20456941 10.1016/j.sbi.2010.03.011 PMC2916977

[7] Martin R. , Straub A.U., Doebele C., Bohnsack M.T. DExD/H-box RNA helicases in ribosome biogenesis. RNA Biol. 2013; 10:4–18. 22922795 10.4161/rna.21879 PMC3590236

[8] Rodríguez-Galán O. , García-Gómez J.J., de la Cruz J. Yeast and human RNA helicases involved in ribosome biogenesis: current status and perspectives. Biochim. Biophys. Acta. 2013; 1829:775–790. 23357782 10.1016/j.bbagrm.2013.01.007

[9] Aquino G.R.R. , Krogh N., Hackert P., Martin R., Gallesio J.D., van Nues R.W., Schneider C., Watkins N.J., Nielsen H., Bohnsack K.E. et al. RNA helicase-mediated regulation of snoRNP dynamics on pre-ribosomes and rRNA 2′-O-methylation. Nucleic Acids Res. 2021; 49:4066–4084. 33721027 10.1093/nar/gkab159 PMC8053091

[10] Bohnsack M.T. , Martin R., Granneman S., Ruprecht M., Schleiff E., Tollervey D Prp43 bound at different sites on the pre-rRNA performs distinct functions in ribosome synthesis. Mol. Cell. 2009; 36:583–592. 19941819 10.1016/j.molcel.2009.09.039 PMC2806949

[11] Bruning L. , Hackert P., Martin R., Davila Gallesio J., Aquino G.R.R., Urlaub H., Sloan K.E., Bohnsack M.T. RNA helicases mediate structural transitions and compositional changes in pre-ribosomal complexes. Nat. Commun. 2018; 9:5383. 30568249 10.1038/s41467-018-07783-w PMC6300602

[12] Jaafar M. , Contreras J., Dominique C., Martin-Villanueva S., Capeyrou R., Vitali P., Rodriguez-Galan O., Velasco C., Humbert O., Watkins N.J. et al. Association of snR190 snoRNA chaperone with early pre-60S particles is regulated by the RNA helicase Dbp7 in yeast. Nat. Commun. 2021; 12:6153. 34686656 10.1038/s41467-021-26207-w PMC8536666

[13] Liang X.H. , Fournier M.J. The helicase Has1p is required for snoRNA release from pre-rRNA. Mol. Cell. Biol. 2006; 26:7437–7450. 16908538 10.1128/MCB.00664-06 PMC1636851

[14] Martin R. , Hackert P., Ruprecht M., Simm S., Brüning L., Mirus O., Sloan K.E., Kudla G., Schleiff E., Bohnsack M.T. A pre-ribosomal RNA interaction network involving snoRNAs and the Rok1 helicase. RNA. 2014; 20:1173–1182. 24947498 10.1261/rna.044669.114 PMC4105744

[15] Sardana R. , Liu X., Granneman S., Zhu J., Gill M., Papoulas O., Marcotte E.M., Tollervey D., Correll C.C., Johnson A.W. The DEAH-box helicase Dhr1 dissociates U3 from the pre-rRNA to promote formation of the central pseudoknot. PLoS Biol. 2015; 13:e1002083. 25710520 10.1371/journal.pbio.1002083 PMC4340053

[16] Aquino G.R.R. , Hackert P., Krogh N., Pan K.T., Jaafar M., Henras A.K., Nielsen H., Urlaub H., Bohnsack K.E., Bohnsack M.T. The RNA helicase Dbp7 promotes domain V/VI compaction and stabilization of inter-domain interactions during early 60S assembly. Nat. Commun. 2021; 12:6152. 34686661 10.1038/s41467-021-26208-9 PMC8536713

[17] Gorbalenya A.E. , Koonin E.V., Donchenko A.P., Blinov V.M. A conserved NTP-motif in putative helicases. Nature. 1988; 333:22. 10.1038/333022a0 2834648

[18] Linder P. , Jankowsky E. From unwinding to clamping - the DEAD box RNA helicase family. *Nature reviews*. Mol. Cell Biol. 2011; 12:505–516. 10.1038/nrm3154 21779027

[19] Putnam A.A. , Jankowsky E. DEAD-box helicases as integrators of RNA, nucleotide and protein binding. Biochim. Biophys. Acta. 2013; 1829:884–893. 23416748 10.1016/j.bbagrm.2013.02.002 PMC3661757

[20] Bono F. , Ebert J., Lorentzen E., Conti E. The crystal structure of the exon junction complex reveals how it maintains a stable grip on mRNA. Cell. 2006; 126:713–725. 16923391 10.1016/j.cell.2006.08.006

[21] Liu F. , Putnam A.A., Jankowsky E. DEAD-box helicases form nucleotide-dependent, long-lived complexes with RNA. Biochemistry. 2014; 53:423–433. 24367975 10.1021/bi401540q

[22] Nielsen K.H. , Chamieh H., Andersen C.B., Fredslund F., Hamborg K., Le Hir H., Andersen G.R. Mechanism of ATP turnover inhibition in the EJC. RNA. 2009; 15:67–75. 19033377 10.1261/rna.1283109 PMC2612766

[23] Kim J.L. , Morgenstern K.A., Griffith J.P., Dwyer M.D., Thomson J.A., Murcko M.A., Lin C., Caron P.R. Hepatitis C virus NS3 RNA helicase domain with a bound oligonucleotide: the crystal structure provides insights into the mode of unwinding. Structure. 1998; 6:89–100. 9493270 10.1016/s0969-2126(98)00010-0

[24] Korolev S. , Yao N., Lohman T.M., Weber P.C., Waksman G. Comparisons between the structures of HCV and Rep helicases reveal structural similarities between SF1 and SF2 super-families of helicases. Protein Sci. 1998; 7:605–610. 9541392 10.1002/pro.5560070309 PMC2143965

[25] Mallam A.L. , Del Campo M., Gilman B., Sidote D.J., Lambowitz A.M. Structural basis for RNA-duplex recognition and unwinding by the DEAD-box helicase Mss116p. Nature. 2012; 490:121–125. 22940866 10.1038/nature11402 PMC3465527

[26] Mallam A.L. , Sidote D.J., Lambowitz A.M. Molecular insights into RNA and DNA helicase evolution from the determinants of specificity for a DEAD-box RNA helicase. Elife. 2014; 3:e04630. 25497230 10.7554/eLife.04630 PMC4383044

[27] Schütz P. , Karlberg T., van den Berg S., Collins R., Lehtiö L., Högbom M., Holmberg-Schiavone L., Tempel W., Park H.W., Hammarström M. et al. Comparative structural analysis of human DEAD-box RNA helicases. PLoS One. 2010; 5:e12791. 20941364 10.1371/journal.pone.0012791 PMC2948006

[28] Sloan K.E. , Bohnsack M.T. Unravelling the mechanisms of RNA helicase regulation. Trends Biochem. Sci. 2018; 43:237–250. 29486979 10.1016/j.tibs.2018.02.001

[29] Bernstein K.A. , Granneman S., Lee A.V., Manickam S., Baserga S.J. Comprehensive mutational analysis of yeast DEXD/H box RNA helicases involved in large ribosomal subunit biogenesis. Mol. Cell. Biol. 2006; 26:1195–1208. 16449635 10.1128/MCB.26.4.1195-1208.2006 PMC1367183

[30] Rosado I.V. , Dez C., Lebaron S., Caizergues-Ferrer M., Henry Y., de la Cruz J. Characterization of Saccharomyces cerevisiae Npa2p (Urb2p) reveals a low-molecular-mass complex containing Dbp6p, Npa1p (Urb1p), Nop8p, and Rsa3p involved in early steps of 60S ribosomal subunit biogenesis. Mol. Cell. Biol. 2007; 27:1207–1221. 17145778 10.1128/MCB.01523-06 PMC1800719

[31] Ismail S. , Flemming D., Thoms M., Gomes-Filho J.V., Randau L., Beckmann R., Hurt E. Emergence of the primordial pre-60S from the 90S pre-ribosome. Cell Rep. 2022; 39:110640. 35385737 10.1016/j.celrep.2022.110640 PMC8994135

[32] Pratte D. , Singh U., Murat G., Kressler D Mak5 and Ebp2 act together on early pre-60S particles and their reduced functionality bypasses the requirement for the essential pre-60S factor Nsa1. PLoS One. 2013; 8:e82741. 24312670 10.1371/journal.pone.0082741 PMC3846774

[33] Burlacu E. , Lackmann F., Aguilar L.C., Belikov S., Nues R.V., Trahan C., Hector R.D., Dominelli-Whiteley N., Cockroft S.L., Wieslander L. et al. High-throughput RNA structure probing reveals critical folding events during early 60S ribosome assembly in yeast. Nat. Commun. 2017; 8:714. 28959008 10.1038/s41467-017-00761-8 PMC5620067

[34] Gamalinda M. , Ohmayer U., Jakovljevic J., Kumcuoglu B., Woolford J., Mbom B., Lin L., Woolford J.L. Jr A hierarchical model for assembly of eukaryotic 60S ribosomal subunit domains. Genes Dev. 2014; 28:198–210. 24449272 10.1101/gad.228825.113 PMC3909792

[35] Kressler D. , de la Cruz J., Rojo M., Linder P. Dbp6p is an essential putative ATP-dependent RNA helicase required for 60S-ribosomal-subunit assembly in Saccharomyces cerevisiae. Mol. Cell. Biol. 1998; 18:1855–1865. 9528757 10.1128/mcb.18.4.1855 PMC121415

[36] Joret C. , Capeyrou R., Belhabich-Baumas K., Plisson-Chastang C., Ghandour R., Humbert O., Fribourg S., Leulliot N., Lebaron S., Henras A.K. et al. The Npa1p complex chaperones the assembly of the earliest eukaryotic large ribosomal subunit precursor. PLos Genet. 2018; 14:e1007597. 30169518 10.1371/journal.pgen.1007597 PMC6136799

[37] Henras A. , Dez C., Noaillac-Depeyre J., Henry Y., Caizergues-Ferrer M. Accumulation of H/ACA snoRNPs depends on the integrity of the conserved central domain of the RNA-binding protein Nhp2p. Nucleic Acids Res. 2001; 29:2733–2746. 11433018 10.1093/nar/29.13.2733 PMC55775

[38] Granneman S. , Kudla G., Petfalski E., Tollervey D Identification of protein binding sites on U3 snoRNA and pre-rRNA by UV cross-linking and high-throughput analysis of cDNAs. Proc. Natl. Acad. Sci. U.S.A. 2009; 106:9613–9618. 19482942 10.1073/pnas.0901997106 PMC2688437

[39] Tollervey D. A yeast small nuclear RNA is required for normal processing of pre-ribosomal RNA. EMBO J. 1987; 6:4169–4175. 3327689 10.1002/j.1460-2075.1987.tb02763.x PMC553900

[40] Lebaron S. , Papin C., Capeyrou R., Chen Y.L., Froment C., Monsarrat B., Caizergues-Ferrer M., Grigoriev M., Henry Y. The ATPase and helicase activities of Prp43p are stimulated by the G-patch protein Pfa1p during yeast ribosome biogenesis. EMBO J. 2009; 28:3808–3819. 19927118 10.1038/emboj.2009.335 PMC2797057

[41] Mouffok S. , Capeyrou R., Belhabich-Baumas K., Joret C., Henras A.K., Humbert O., Henry Y. The G-patch activators Pfa1 and PINX1 exhibit different modes of interaction with the Prp43 RNA helicase. RNA Biol. 2021; 18:510–522. 32882145 10.1080/15476286.2020.1818458 PMC7971346

[42] Putnam A.A. , Gao Z., Liu F., Jia H., Yang Q., Jankowsky E. Division of labor in an oligomer of the DEAD-box RNA helicase Ded1p. Mol. Cell. 2015; 59:541–552. 26212457 10.1016/j.molcel.2015.06.030 PMC4546514

[43] Bizebard T. , Ferlenghi I., Iost I., Dreyfus M. Studies on three *E. coli* DEAD-box helicases point to an unwinding mechanism different from that of model DNA helicases. Biochemistry. 2004; 43:7857–7866. 15196029 10.1021/bi049852s

[44] Kim Y. , Myong S. RNA remodeling activity of DEAD box proteins tuned by protein concentration, RNA length, and ATP. Mol. Cell. 2016; 63:865–876. 27546789 10.1016/j.molcel.2016.07.010 PMC5010468

[45] Tijerina P. , Bhaskaran H., Russell R. Nonspecific binding to structured RNA and preferential unwinding of an exposed helix by the CYT-19 protein, a DEAD-box RNA chaperone. Proc. Natl. Acad. Sci. U.S.A. 2006; 103:16698–16703. 17075070 10.1073/pnas.0603127103 PMC1636518

[46] Young C.L. , Khoshnevis S., Karbstein K. Cofactor-dependent specificity of a DEAD-box protein. Proc. Natl. Acad. Sci. U.S.A. 2013; 110:E2668–E2676. 23630256 10.1073/pnas.1302577110 PMC3718167

[47] Halls C. , Mohr S., Del Campo M., Yang Q., Jankowsky E., Lambowitz A.M. Involvement of DEAD-box proteins in group I and group II intron splicing. Biochemical characterization of Mss116p, ATP hydrolysis-dependent and -independent mechanisms, and general RNA chaperone activity. J. Mol. Biol. 2007; 365:835–855. 17081564 10.1016/j.jmb.2006.09.083 PMC1832103

[48] Yang Q. , Jankowsky E. ATP- and ADP-dependent modulation of RNA unwinding and strand annealing activities by the DEAD-box protein DED1. Biochemistry. 2005; 44:13591–13601. 16216083 10.1021/bi0508946

[49] Jarmoskaite I. , Russell R. RNA helicase proteins as chaperones and remodelers. Annu. Rev. Biochem. 2014; 83:697–725. 24635478 10.1146/annurev-biochem-060713-035546 PMC4143424

[50] Daugeron M.C. , Kressler D., Linder P. Dbp9p, a putative ATP-dependent RNA helicase involved in 60S-ribosomal-subunit biogenesis, functionally interacts with Dbp6p. RNA. 2001; 7:1317–1334. 11565753 10.1017/s1355838201010640 PMC1370175

[51] Henn A. , Bradley M.J., De La Cruz E.M. ATP utilization and RNA conformational rearrangement by DEAD-box proteins. Annu. Rev. Biophys. 2012; 41:247–267. 22404686 10.1146/annurev-biophys-050511-102243 PMC7761782

[52] Ozgur S. , Buchwald G., Falk S., Chakrabarti S., Prabu J.R., Conti E. The conformational plasticity of eukaryotic RNA-dependent ATPases. FEBS J. 2015; 282:850–863. 25645110 10.1111/febs.13198

[53] Del Campo M. , Mohr S., Jiang Y., Jia H., Jankowsky E., Lambowitz A.M. Unwinding by local strand separation is critical for the function of DEAD-box proteins as RNA chaperones. J. Mol. Biol. 2009; 389:674–693. 19393667 10.1016/j.jmb.2009.04.043 PMC2769564

[54] Kelley L.A. , Mezulis S., Yates C.M., Wass M.N., Sternberg M.J.E. The Phyre2 web portal for protein modeling, prediction and analysis. Nat. Protoc. 2015; 10:845–858. 25950237 10.1038/nprot.2015.053 PMC5298202

[55] Yang J. , Yan R., Roy A., Xu D., Poisson J., Zhang Y. The I-TASSER Suite: protein structure and function prediction. Nat. Methods. 2015; 12:7–8. 10.1038/nmeth.3213 PMC4428668 25549265

[56] Xue B. , Dunbrack R.L., Williams R.W., Dunker A.K., Uversky V.N. PONDR-FIT: a meta-predictor of intrinsically disordered amino acids. Biochim. Biophys. Acta. 2010; 1804:996–1010. 20100603 10.1016/j.bbapap.2010.01.011 PMC2882806

[57] Chou C.C. , Wang A.H. Structural D/E-rich repeats play multiple roles especially in gene regulation through DNA/RNA mimicry. Mol. Biosyst. 2015; 11:2144–2151. 26088262 10.1039/c5mb00206k

[58] Yoshikawa H. , Larance M., Harney D.J., Sundaramoorthy R., Ly T., Owen-Hughes T., Lamond A.I. Efficient analysis of mammalian polysomes in cells and tissues using Ribo Mega-SEC. Elife. 2018; 7:e36530. 30095066 10.7554/eLife.36530 PMC6086667

[59] Cordin O. , Banroques J., Tanner N.K., Linder P. The DEAD-box protein family of RNA helicases. Gene. 2006; 367:17–37. 16337753 10.1016/j.gene.2005.10.019

[60] Gnanasundram S.V. , Kos-Braun I.C., Koš M. At least two molecules of the RNA helicase Has1 are simultaneously present in pre-ribosomes during ribosome biogenesis. Nucleic Acids Res. 2019; 47:10852–10864. 31511893 10.1093/nar/gkz767 PMC6846684

[61] Lebaron S. , Segerstolpe A., French S.L., Dudnakova T., de Lima Alves F., Granneman S., Rappsilber J., Beyer A.L., Wieslander L., Tollervey D Rrp5 binding at multiple sites coordinates pre-rRNA processing and assembly. Mol. Cell. 2013; 52:707–719. 24239293 10.1016/j.molcel.2013.10.017 PMC3991325

[62] Manikas R.G. , Thomson E., Thoms M., Hurt E. The K^+^-dependent GTPase Nug1 is implicated in the association of the helicase Dbp10 to the immature peptidyl transferase centre during ribosome maturation. Nucleic Acids Res. 2016; 44:1800–1812. 26823502 10.1093/nar/gkw045 PMC4770245

[63] Bohnsack M.T. , Kos M., Tollervey D Quantitative analysis of snoRNA association with pre-ribosomes and release of snR30 by Rok1 helicase. EMBO Rep. 2008; 9:1230–1236. 18833290 10.1038/embor.2008.184 PMC2570499

[64] Choudhury P. , Hackert P., Memet I., Sloan K.E., Bohnsack M.T. The human RNA helicase DHX37 is required for release of the U3 snoRNP from pre-ribosomal particles. RNA Biol. 2019; 16:54–68. 30582406 10.1080/15476286.2018.1556149 PMC6380342

[65] Kater L. , Thoms M., Barrio-Garcia C., Cheng J., Ismail S., Ahmed Y.L., Bange G., Kressler D., Berninghausen O., Sinning I. et al. Visualizing the assembly pathway of nucleolar pre-60S ribosomes. Cell. 2017; 171:1599–1610. 29245012 10.1016/j.cell.2017.11.039 PMC5745149

[66] Baßler J. , Hurt E. Eukaryotic ribosome assembly. Annu. Rev. Biochem. 2019; 88:281–306. 30566372 10.1146/annurev-biochem-013118-110817

[67] Sanghai Z.A. , Miller L., Molloy K.R., Barandun J., Hunziker M., Chaker-Margot M., Wang J., Chait B.T., Klinge S. Modular assembly of the nucleolar pre-60S ribosomal subunit. Nature. 2018; 556:126–129. 29512650 10.1038/nature26156 PMC6118127

[68] Zhou D. , Zhu X., Zheng S., Tan D., Dong M.Q., Ye K. Cryo-EM structure of an early precursor of large ribosomal subunit reveals a half-assembled intermediate. Protein Cell. 2019; 10:120–130. 29557065 10.1007/s13238-018-0526-7 PMC6340896

[69] Dez C. , Froment C., Noaillac-Depeyre J., Monsarrat B., Caizergues-Ferrer M., Henry Y. Npa1p, a component of very early pre-60S ribosomal particles, associates with a subset of small nucleolar RNPs required for peptidyl transferase center modification. Mol. Cell. Biol. 2004; 24:6324–6337. 15226434 10.1128/MCB.24.14.6324-6337.2004 PMC434229

[70] Bhaskaran H. , Russell R. Kinetic redistribution of native and misfolded RNAs by a DEAD-box chaperone. Nature. 2007; 449:1014–1018. 17960235 10.1038/nature06235 PMC2581903

[71] Andreou A.Z. , Harms U., Klostermeier D eIF4B stimulates eIF4A ATPase and unwinding activities by direct interaction through its 7-repeats region. RNA Biol. 2017; 14:113–123. 27858515 10.1080/15476286.2016.1259782 PMC5270515

[72] Hilbert M. , Kebbel F., Gubaev A., Klostermeier D eIF4G stimulates the activity of the DEAD box protein eIF4A by a conformational guidance mechanism. Nucleic Acids Res. 2011; 39:2260–2270. 21062831 10.1093/nar/gkq1127 PMC3064780

[73] Rogers G.W. Jr , Richter N.J., Merrick W.C Biochemical and kinetic characterization of the RNA helicase activity of eukaryotic initiation factor 4A. J. Biol. Chem. 1999; 274:12236–12244. 10212190 10.1074/jbc.274.18.12236

[74] Schütz P. , Bumann M., Oberholzer A.E., Bieniossek C., Trachsel H., Altmann M., Baumann U. Crystal structure of the yeast eIF4A-eIF4G complex: an RNA-helicase controlled by protein-protein interactions. Proc. Natl. Acad. Sci. U.S.A. 2008; 105:9564–9569. 18606994 10.1073/pnas.0800418105 PMC2474498

[75] Garcia I. , Albring M.J., Uhlenbeck O.C. Duplex destabilization by four ribosomal DEAD-box proteins. Biochemistry. 2012; 51:10109–10118. 23153376 10.1021/bi301172s

[76] Xiol J. , Spinelli P., Laussmann M.A., Homolka D., Yang Z., Cora E., Couté Y., Conn S., Kadlec J., Sachidanandam R. et al. RNA clamping by Vasa assembles a piRNA amplifier complex on transposon transcripts. Cell. 2014; 157:1698–1711. 24910301 10.1016/j.cell.2014.05.018

