# Supplementary material for: The DEAD-box protein Dbp6 is an ATPase and RNA annealase interacting with the peptidyl transferase center (PTC) of the ribosome
Source: Nucleic Acids Res. 2023 Jan 5;51(2):744–64. doi: 10.1093/nar/gkac1196 (PMC9881158; doi:10.1093/nar/gkac1196)
Supplement: gkac1196_Supplemental_File [file gkac1196_supplemental_file.pdf]

SUPPLEMENTARY INFORMATIONS

Supplementary materials and methods

Yeast strains and media

Strains expressing WT and mutant GFP-tagged Dbp6 under the control of an oestradiol-regulated promoter have been obtained by transforming yeast W303 (*leu2-3,112 trp1-1 can1-100 ura3-1 ade2-1 his3-11,15*) with plasmids constructed with the Gateway™ Cloning Technology (Invitrogen) using WT and mutant Dbp6 cassettes that were introduced in the vector pAGA13GAL-ccdB-EGFP (Addgene plasmid # 14405; http://n2t.net/addgene:14190; RRID:Addgene_14190). Transformed strains have been selected on YNB medium lacking histidine. Strains have been grown in YNB (6.8 g/L) supplemented with ammonium sulphate (20 g/L), the required amino acids and 2% glucose.

Unwinding assays

For the unwinding assay of Fig. S2B, Dbp6 was pre-incubated for 30 min at 30°C with 1 nM of labeled substrate (hemi-duplex ds12/30, i.e. ss30-mer hybridized to radiolabelled ss12-mer) and 50 µM of ATP or ADP, when indicated, in RB 1X (2.5 mM Tris-HCl pH 8, 2.5 mM $MgCl_2$, 10 mM KCl, 0.02 mM DTT, 10 mg/mL BSA) supplemented with 0.03 units of RNasin (Promega). The reaction was pursued for a further 8 min after addition or not of 1 mM ATP. The reaction was then stopped with Stop buffer containing 2 µM of trap oligonucleotide (Comp-58) that is complementary to the ss30-mer and used to prevent reannealing of the radiolabelled ss12-mer oligonucleotide. For the unwinding assay of Fig. S2C, the reaction was carried out as described for Fig. 1D, without pre-incubation. The substrate (1 nM) was a hemi-duplex ds9/30 (ss30-mer hybridized to a radiolabelled ss9-mer).

Ribo Mega-SEC

Ribo Mega-SEC experiments were adapted from (1). Briefly, yeast cells were grown to an OD_{600nm} ~0.6-0.8, incubated with a final concentration of 50 µg/ml of cycloheximide for 10 min, collected by centrifugation followed by two washes with the TMK buffer (20 mM Tris-HCl pH 7.4, 50 mM KCl and 10 mM $MgCl_2$) supplemented with 50 µg/ml of cycloheximide and centrifugation. Yeast pellets were then frozen at -80°C. Cells were thawed and resuspended with extraction buffer (20 mM Hepes-NaOH pH 7.4, 130 mM NaCl, 10 mM $MgCl_2$, 1% CHAPS, 0.2 mg/ml heparin, 2.5 mM DTT, 50 µg/ml cycloheximide and 0.1 units/µl RNasin (Promega)) supplemented with complete EDTA-free protease inhibitor (Roche). Cells were broken by adding two volumes of Zicornia beads and performing 5 runs of 1 min of vigorous vortexing with 1 min of incubation in ice in-between each run. Cell extracts were clarified twice by 10 min centrifugation at 16000xg and quantified by NanoDrop (A_{260}, NP80 Implen). Aliquots of clarified extracts (50 µl at 2 µg/µl) were injected at a 0.2 ml/min flow rate in a 2000 Å Bio

SEC-5 gel filtration column (Agilent) equilibrated with SEC buffer (20 mM Hepes-NaOH pH 7.4, 60 mM NaCl, 10 mM $MgCl_2$, 0.3% CHAPS, 0.2 mg/ml heparin and 2.5 mM DTT). 250 µl fractions were collected that were frozen at -20°C. The samples were then analyzed by western and northern blots to check protein and RNA levels, respectively.

Supplementary figure and table legends

Figure S1: Purification of recombinant Dbp6

A) Chromatogram from gel filtration on Superdex S-200 column of Dbp6. The major exclusion peak at 70.24 ml of elution volume is expected for a 146 kDa protein, estimated from molecular weight standards, and is consistent here with a mix of dimeric and monomeric (end of the peak) Dbp6. Fractions covering the peak were annotated F14 to F18. On the volume axis, s1 to s7 mark the elution volumes of the molecular weight standards used for column calibration (s1: Blue Dextran, 2000 kDa; s2: Ferritin, 440 kDa; s3: Aldolase, 158 kDa; s4: BSA, 67 kDa; s5: Ovalbumine, 43 kDa; s6: Carbonic anhydrase, 29 kDa; s7: Ribonuclease A, 13.7 kDa). *mAU*, milliarbitrary unit. **B**) Coomassie blue staining of oligomeric fractions (F5-F9, lanes 3-7) and di-monomeric fractions (F14-F18, lanes 8-12). Lane 1: Input Dbp6 from nickel purification loaded on Superdex S-200 column; lane 2: Molecular weight markers. **C**) Western blot analysis of fractions F14 to F18 (lanes 1 to 5) and fraction F23 (lane 6), using polyclonal anti-Dbp6 antibodies. **D)** Analysis of Dbp6 oligomeric state in key size-exclusion fractions. After UV cross-linking, proteins present in F15, F17 and F18 peak fractions and in high molecular weight F8 fraction ("oligo") were separated by SDS-PAGE and the different forms of Dbp6 were detected by western blot using polyclonal anti-Dbp6 antibodies (lanes 1 to 4) or visualized by silver staining (lanes 5 to 8). **E**) ATPase assays with Dbp6 monomers (*i.e.* fraction F17) and oligomers (*i.e.* F8). Error bars represent the standard error of the mean (SEM).

Figure S2: Biochemical properties of Dbp6

A) Binding activity on different RNA substrates. Oligonucleotides of different lengths (58-, 38-, or 21-mer), either single- (ss) or double- (ds) stranded, have been tested for complex formation by increasing the amount of Dbp6 (from 0 to 1.2 µM). The ds38/58 substrate corresponds to a hemi-duplex RNA, with a 20 nt 3'-ss overhang, resulting from the annealing of the two mentioned single-stranded oligonucleotides. Plot was computed with GraphPad Prism. Error bars represent SEM for $n \geq 3$ experiments. **B and C)** Assays for unwinding activity. **B)** A scheme of the reaction with a pre-incubation step is given on the left panel, where Dbp6 and the hemi-duplex (ds12/30) RNA are pre-incubated during 30 min to form a clamp, in the presence or absence of nucleotide (ATP or ADP). The reaction is pursued for a further 8 min after addition or not of ATP. The proteinase K-treated products of the reaction (hemi-duplex and free oligonucleotide, schematized on the right of the gel) are separated by non-denaturing PAGE. The percentage of unwinding corresponds to the amount of free oligonucleotide versus the total

amount of RNA. **C)** A ds9/30 substrate that features a 9 base pair duplex part and a 21 nucleotide-long 3' single stranded extension was used in classical helicase assays. On the left panel, the reaction was carried out either with increasing concentration of Dbp6 protein (0.4, 0.6, 0.8, 1 µM) in the presence of ATP (1 mM) (lanes 3, 4, 5, 6) or with 1 µM of protein in the absence of ATP (lane 7), or in the presence of ADP (1 mM) (lane 8). On the right panel, the unwinding capacity of Dbp6 (0.8 µM) was tested in a time course reaction performed in the presence of 1 mM of ATP (lanes 3-8) or ADP (lanes 9 and 10). For both gels, lanes 1 and 2 have been loaded with the free 9-mer oligonucleotide and the hemi-duplex, respectively.

Figure S3: Tests associated to the Dbp6 annealing activity

A) Test of ss38-mer self-association. Increasing amounts of biotinylated ss38-mer oligonucleotide (ss38-Biot), as indicated, were mixed for 10 min at 30°C with radiolabelled ss38-mer oligonucleotide (ss38*, 10 nM), either without (lanes 1-4) or with (lanes 5-7) Dbp6 protein (0.8 µM). Lanes 8 and 9 show the migration of the ds38 duplex (ds38*) formed by hybridization of the radioactive ss38-mer oligonucleotide and its complementary oligonucleotide, in the absence or presence of Dbp6. After proteinase K treatment, all the RNA species were separated by non-denaturing PAGE. **B)** Assay for Dbp6-Dbp6 interactions *in vivo*. Precipitation experiments have been carried out with IgG-sepharose and RNase-treated extracts from cells expressing HA-tagged Dbp6 (HA-Dbp6) from the endogenous chromosomal locus transformed with a plasmid (pWT) directing expression of ZZ-tagged Dbp6 (Dbp6-ZZ) or with an empty control plasmid (pEmpty). Dbp6-ZZ and HA-Dbp6 present in aliquots of initial input (IP), flow through (FT) and precipitated (IP) samples were detected by western blot with anti-Dbp6 antibodies. The histogram shows the co-precipitation efficiency of HA-Dbp6 in the presence of Dbp6-ZZ, relative to HA-Dbp6 background precipitation in the absence of Dbp6-ZZ (set at 1). Error bar: standard deviation (SD) for 3 experiments.

Figure S4: Effects of Dbp6 depletion on pre-rRNA processing

GAL::HA-DBP6 cells and parental BY4742 cells were grown in a galactose-containing medium then shifted into a glucose-containing medium. Total proteins and RNAs were extracted from cell samples collected at different times after the shift (0, 3, 6, 9, 12 hours) and analyzed by western (A) and northern (B) blots. **A**) Dbp6 was detected with a specific antibody. Dbp6 levels decrease drastically 3 hours after the shift and remain undetectable afterwards. Pgk1 was used as a loading control. **B**) The 35S and $27SA_2$ pre-rRNAs were detected with the 23S1 probe (top panel). Quantification of the results, presented in the histogram (lower panel), shows an accumulation of the 35S and a decrease of the $27SA_2$ pre-rRNAs 6 hours after the shift.

Figure S5: Effects of increased expression of wild-type Dbp6 or Dbp6 M-II mutant on cell growth

Wild-type W303 strain was transformed with plasmids encoding GFP-tagged Dbp6 (Dbp6-GFP) or GFP-tagged Dbp6 M-II mutant (M-II-GFP) under the control of an oestradiol-regulated promoter, or the empty parental plasmid (control). **A**) The strain transformed with the plasmid encoding Dbp6-GFP was grown in the absence (-) or from 30 to 180 min in the presence of 10 or 100 mM oestradiol. Dbp6-GFP and endogenous Dbp6 levels were determined by western using anti-Dbp6 antibodies. **B**) Transformed strains were grown in liquid YNB medium supplemented or not with 10 or 100 mM oestradiol. Strains were then serially diluted tenfold, spotted on YNB plates containing or not the indicated concentration of oestradiol and incubated at 30ºC for 2.5 days.

Figure S6: Wild-type and mutant Dbp6 elution profile in Ribo Mega-Sec experiment

Extracts from cells expressing wild-type Dbp6 or the indicated Dbp6 mutants (M-II, M-I, ΔN) were fractionated using exclusion chromatography and HPLC. Elution profiles obtained with the wild-type (black) and mutant Dbp6 extracts (M-II, green; M-I, red; ΔN, blue) are shown on the top of the figure. Fractions containing polysomes (F16-20), 80S monosomes (F21-22), 60S and 40S subunits (F23-24) are highlighted. The elution profiles of Dbp6 as well as RPL3 and RPS8 ribosomal proteins, components of the large and small ribosomal subunits, obtained with the wild-type extract were determined by western blot using specific antibodies. The elution profiles of 35S and $27SA_2$ pre-rRNAs and the 18S and 25S mature rRNAs in fractions 19 to 26 obtained with the wild-type extract were determined by northern blot.

Figure S7: Purification of recombinant mutant Dbp6 proteins

A) Top: Superimposed absorption spectra of fractions from gel filtration on Superdex S-200 column of WT (blue) and M-II (green) Dbp6. Bottom: M-II fractions were subjected to SDS-PAGE followed by Coomassie blue staining. **B)** Top: Superimposed absorption spectra of fractions from gel filtration on Superdex S-200 column of WT (blue) and ΔN (cyan) Dbp6. Bottom: ΔN fractions were subjected to SDS-PAGE followed by Coomassie blue staining. For both (**A**) and (**B**), s1 to s7 mark the elution volumes of molecular weight standards also used in Fig. S1A and "MWt" corresponds to the molecular weight markers also used in Fig. S1B. **C**) Ratios of dimer/monomer and oligomer species obtained for the purifications of the WT, M-II, M-I, M-Ia, M-Ic, and ΔN mutants. The peak area of the absorption spectra was calculated (Unicorn software) for each species and reported on the histogram as a percentage for each protein. Error bars represent SEM for $n \geq 3$ experiments.

Figure S8: Dbp6 RNA binding sites identified by CRAC

CRAC results were obtained from BY4742 cells (control) or cells expressing HTP-tagged Dbp6 (HTP-Dbp6). **A**) The percentage of total mapped reads corresponding to each class of RNA is given for the control and HTP-Dbp6 CRAC samples. The percentage is calculated per million of hittable reads. **B**)

The number of reads (upper graph) and the number of mutations/deletions (lower graph), mapping to each nucleotide of the RDN37 rDNA encoding the 35S pre-rRNA precursor, are plotted for the control (green) and HTP-Dbp6 CRAC (red) experiments. Asterisks indicate common contaminating peaks. A corresponding schematic representation of the pre-rRNA transcript is drawn below, showing the mature 18S (green), 5.8S (light blue) and 25S (dark blue) rRNAs. **C**) The main Dbp6 crosslinking sites (identified by numbers on Fig. 7) are mapped in blue on the 2D structure of the 25S rRNA. Nucleotides with a high mutation/deletion incidence are highlighted by dark coloured dots within the sequence. The complementary sequences of the major snoRNAs bound by Dbp6 (listed in Fig. 7) are indicated in red. As additional information, the Npa1 CRAC sites (2) are shown in green.

Figure S9: snoRNAs associated with Dbp6

A) The snoRNAs are listed as a heat map according to their relative enrichment in the Dbp6 CRAC versus control sample data sets using Genesis (from Graz University of Technology). The number of hits per million in both data sets ("HTP-Dbp6" and "control" column) is indicated for each snoRNA. **B)** Alignment of the reads from the Dbp6 CRAC data sets with the different snoRNA sequences. The number of hits and the number of mutations/deletions for each nucleotide of the snoRNA sequence is plotted per million of mapped reads. Black bars below the base positions indicate the regions of base pairing with the pre-rRNA, green bars and blue bars indicate the consensus H/ACA and C/D boxes, respectively.

Supplementary table S1: DNA oligonucleotides used for plasmid construction

Supplementary table S2: RNA and DNA oligonucleotides used in the biochemical assays

Supplementary table S3: DNA oligonucleotides used for northern blot assays

Supplementary table S4: Plasmids directing Dbp6 expression in yeast

Supplementary references

1. Yoshikawa, H., Larance, M., Harney, D.J., Sundaramoorthy, R., Ly, T., Owen-Hughes, T. and Lamond, A.I. (2018) Efficient analysis of mammalian polysomes in cells and tissues using Ribo Mega-SEC. *Elife*, 7.
2. Joret, C., Capeyrou, R., Belhabich-Baumas, K., Plisson-Chastang, C., Ghandour, R., Humbert, O., Fribourg, S., Leulliot, N., Lebaron, S., Henras, A.K. *et al.* (2018) The Npa1p complex chaperones the assembly of the earliest eukaryotic large ribosomal subunit precursor. *PLoS Genet*, 14, e1007597.

Figure S1

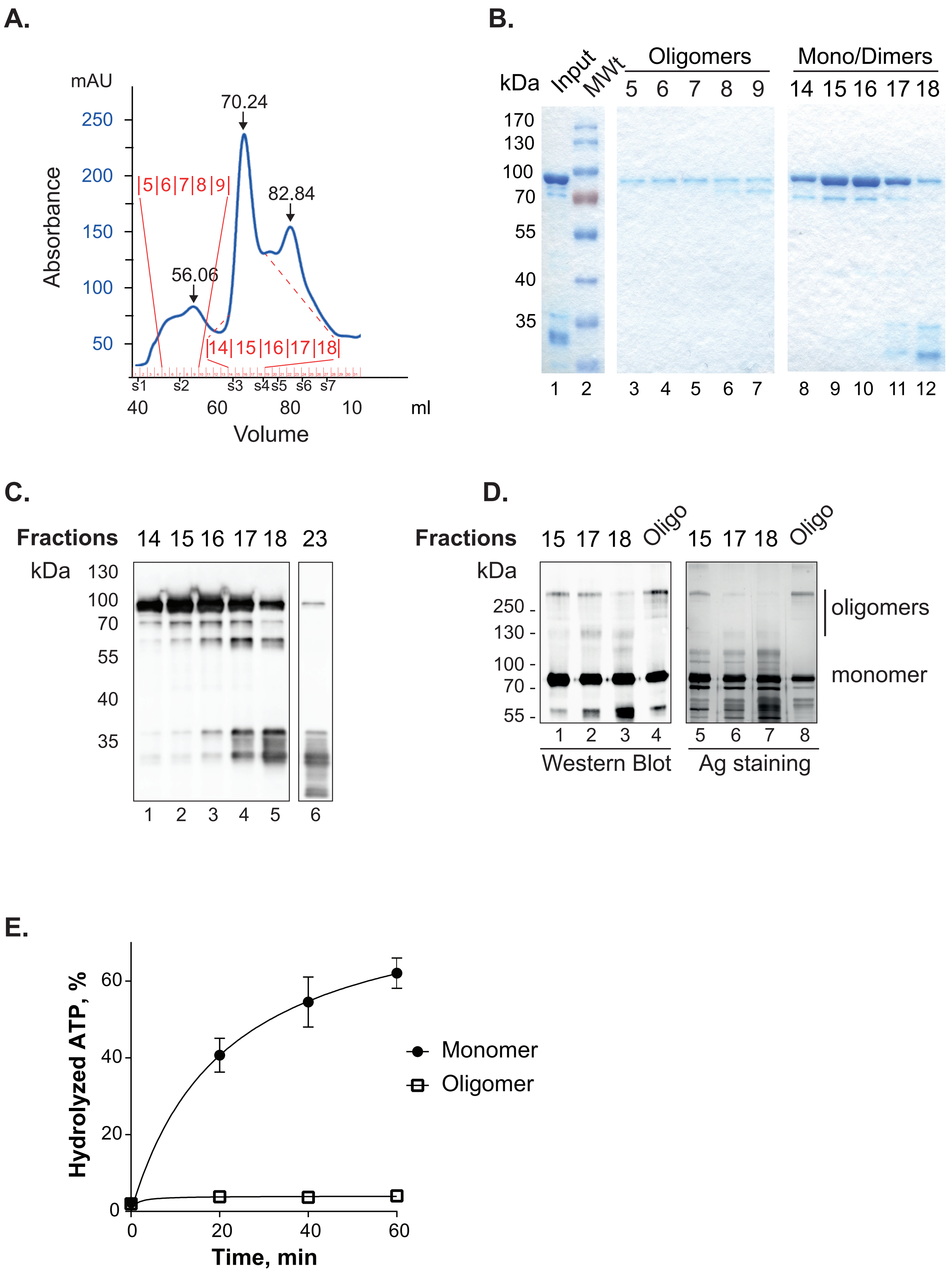

A.
mAU
250
200
150
100
50
Absorbance
70.24
82.84
56.06
|5|6|7|8|9|
|14|15|16|17|18|
s1 s2 s3 s4 s5 s6 s7
40 60 80 10 ml
Volume
B.
kDa
Input
MWt
Oligomers
Mono/Dimers
5 6 7 8 9
14 15 16 17 18
170
130
100
70
55
40
35
1 2 3 4 5 6 7 8 9 10 11 12
C.
Fractions
14 15 16 17 18 23
kDa
130
100
70
55
40
35
1 2 3 4 5 6
D.
Fractions
15 17 18 Oligo 15 17 18 Oligo
kDa
250
130
100
70
55
oligomers
monomer
1 2 3 4 5 6 7 8
Western Blot
Ag staining
E.
Hydrolyzed ATP, %
60
40
20
0
Monomer
Oligomer
0 20 40 60
Time, min

Figure S2

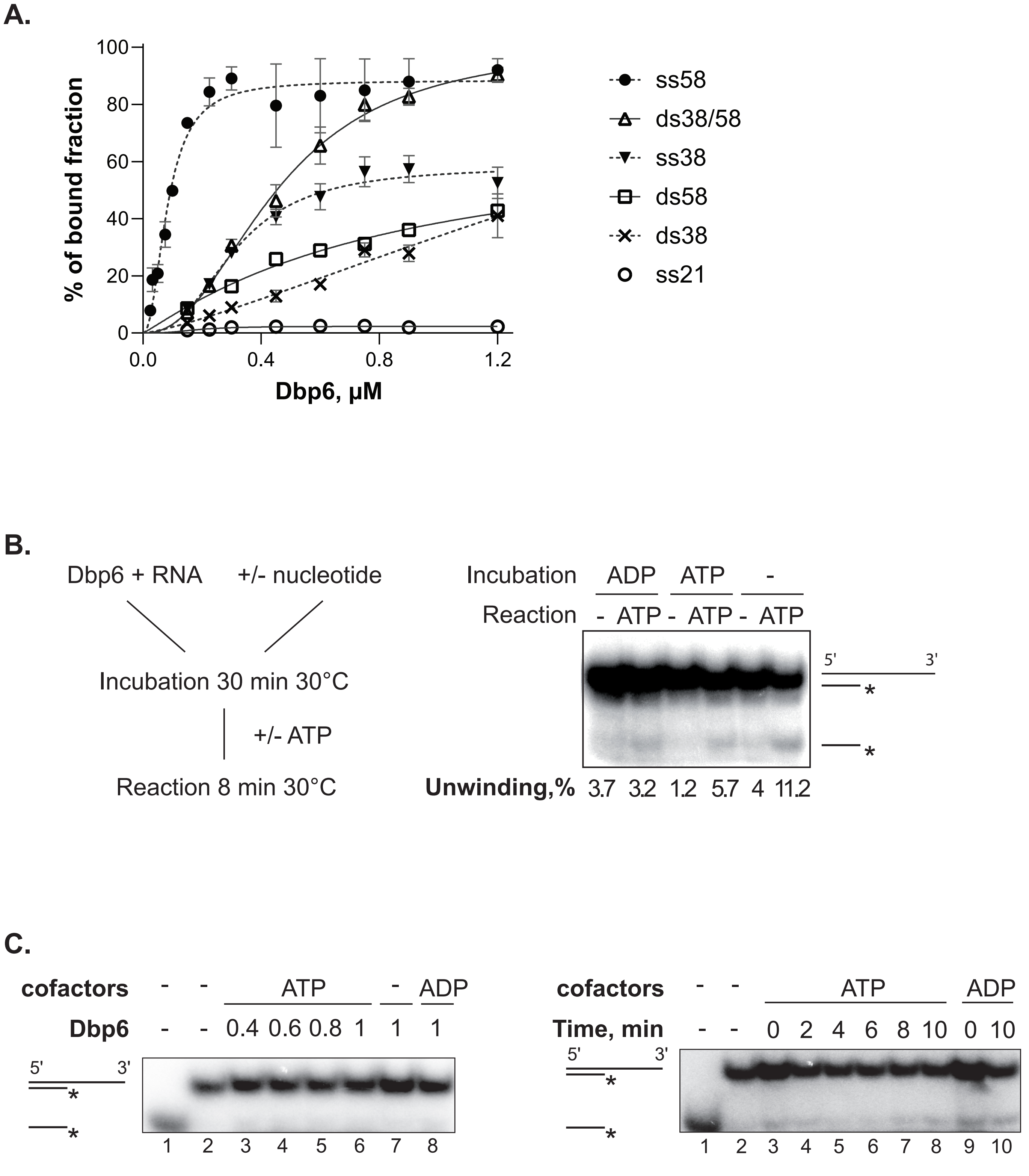
A.
% of bound fraction
Dbp6, µM
ss58
ds38/58
ss38
ds58
ds38
ss21
B.
Dbp6 + RNA
+/- nucleotide
Incubation 30 min 30°C
+/- ATP
Reaction 8 min 30°C
Incubation ADP ATP -
Reaction - ATP - ATP - ATP
Unwinding,% 3.7 3.2 1.2 5.7 4 11.2
C.
cofactors - - ATP - ADP
Dbp6 - - 0.4 0.6 0.8 1 1 1
1 2 3 4 5 6 7 8
cofactors - - ATP ADP
Time, min - - 0 2 4 6 8 10 0 10
1 2 3 4 5 6 7 8 9 10

Figure S3

A.

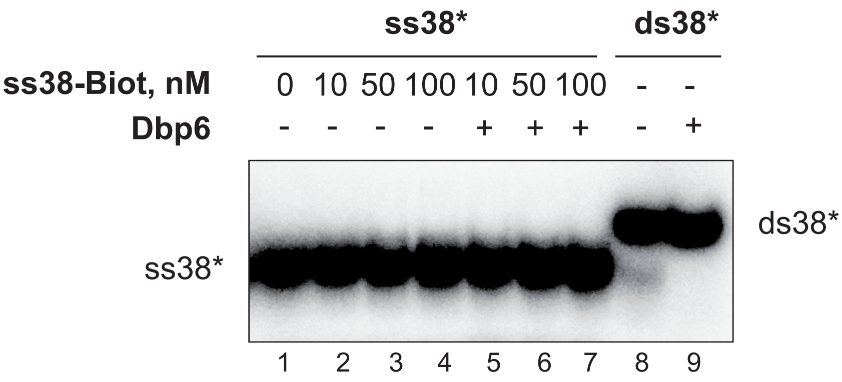

ss38*
ds38*
ss38-Biot, nM
0 10 50 100 10 50 100 - -
Dbp6
- - - - + + + - +
ss38*
ds38*
1 2 3 4 5 6 7 8 9

B.

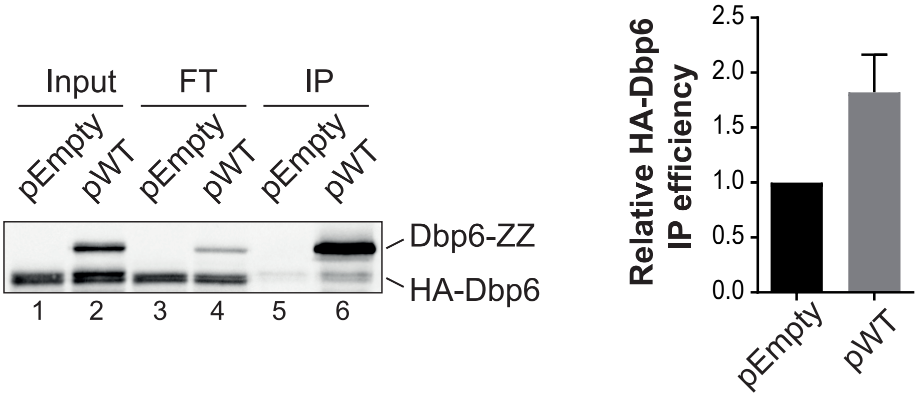

Input
FT
IP
pEmpty
pWT
pEmpty
pWT
pEmpty
pWT
Dbp6-ZZ
HA-Dbp6
1 2 3 4 5 6
Relative HA-Dbp6 IP efficiency
2.5
2.0
1.5
1.0
0.5
0.0
pEmpty
pWT

Figure S4

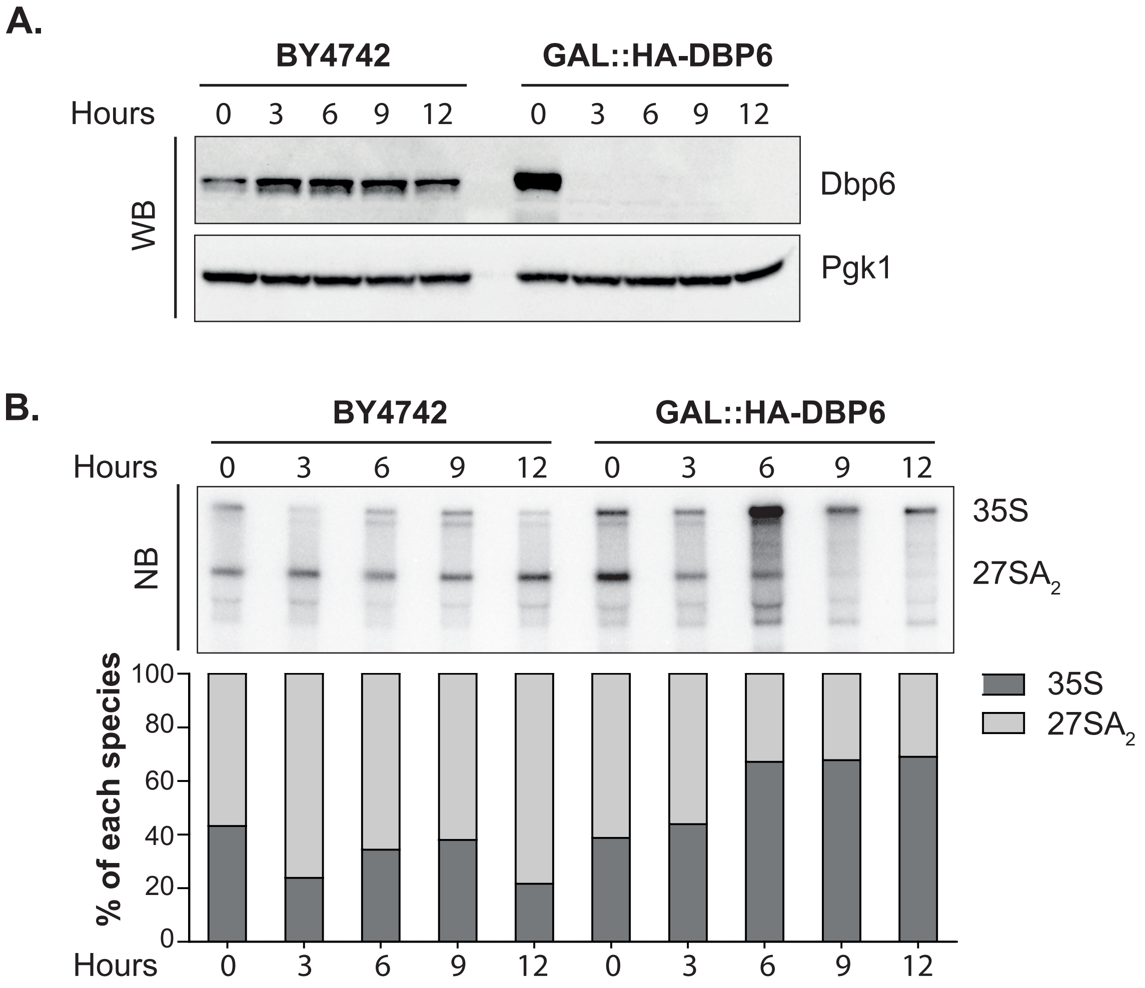
A.
BY4742
GAL::HA-DBP6
Hours
0 3 6 9 12 0 3 6 9 12
WB
Dbp6
Pgk1
B.
BY4742
GAL::HA-DBP6
Hours
0 3 6 9 12 0 3 6 9 12
NB
35S
27SA2
% of each species
100
80
60
40
20
0
Hours
0 3 6 9 12 0 3 6 9 12
35S
27SA2

Figure S5

A.

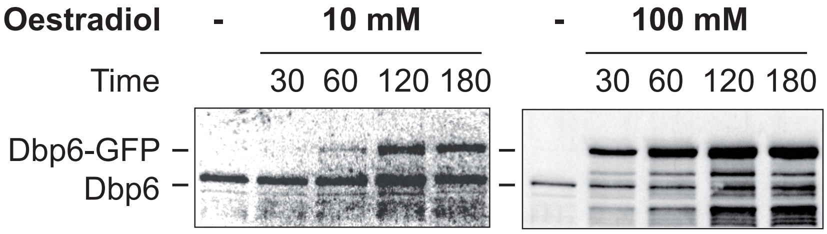
Oestradiol
-
10 mM
-
100 mM
Time
30 60 120 180
30 60 120 180
Dbp6-GFP
Dbp6

B.

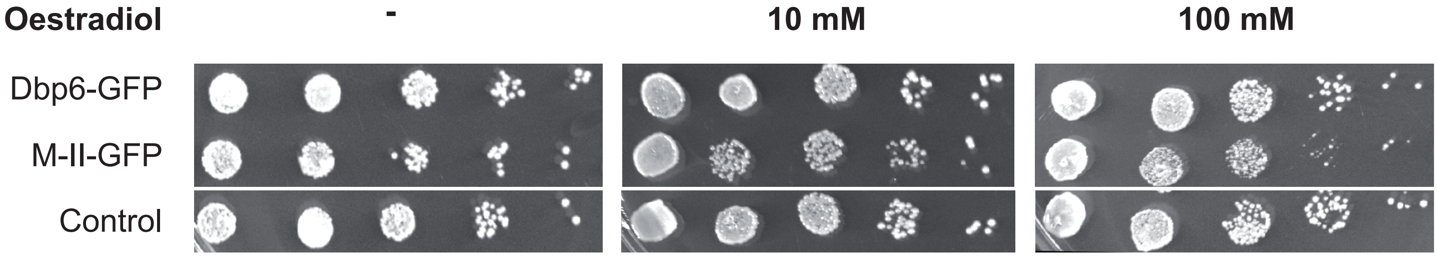
Oestradiol
-
10 mM
100 mM
Dbp6-GFP
M-II-GFP
Control

Figure S6

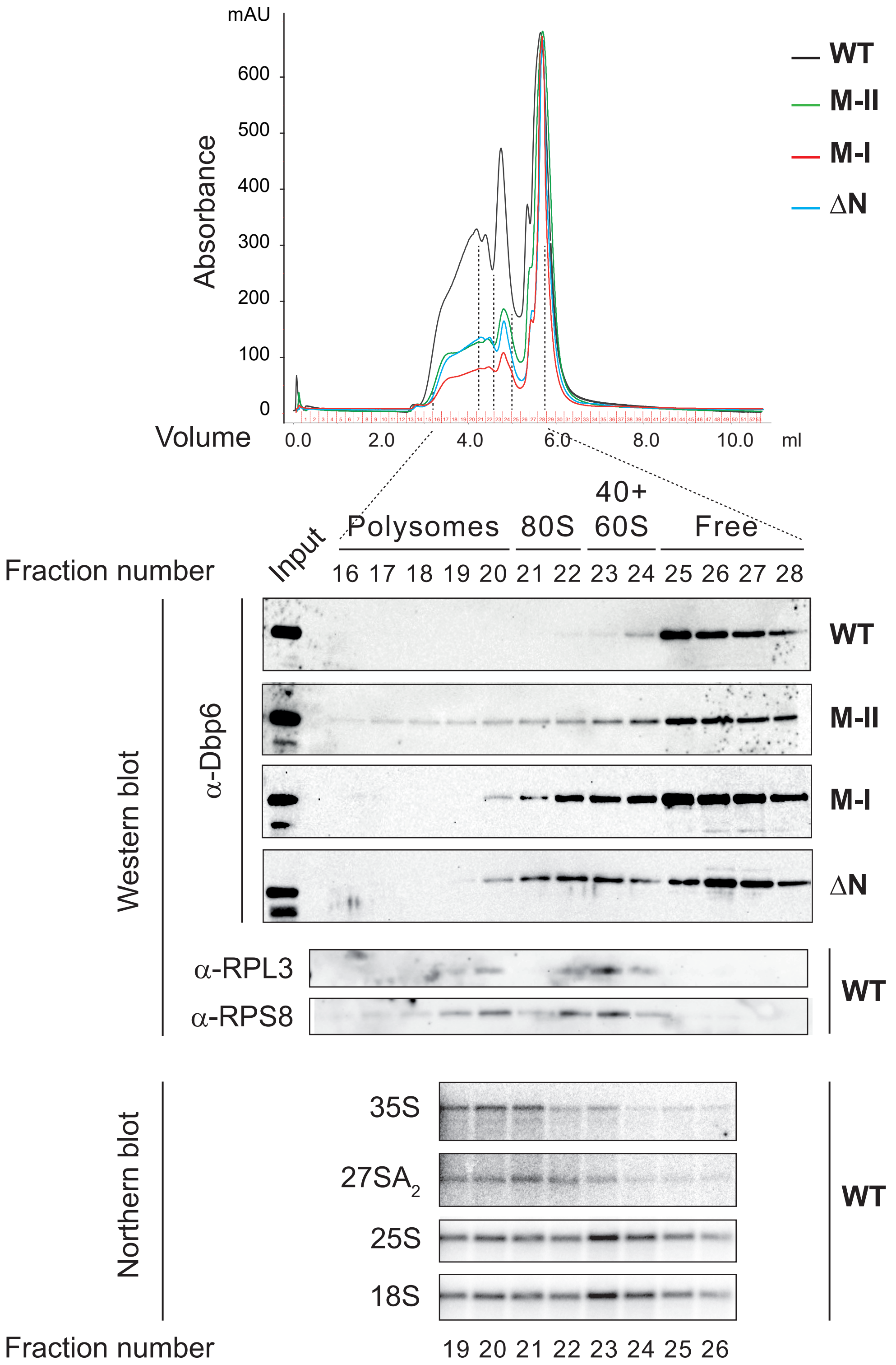

mAU
600
500
400
300
200
100
0
Absorbance
WT
M-II
M-I
ΔN
Volume
0.0
2.0
4.0
6.0
8.0
10.0
ml
Fraction number
Input
Polysomes
80S
40+ 60S
Free
16 17 18 19 20 21 22 23 24 25 26 27 28
Western blot
α-Dbp6
WT
M-II
M-I
ΔN
α-RPL3
α-RPS8
WT
Northern blot
35S
27SA2
25S
18S
WT
Fraction number
19 20 21 22 23 24 25 26

Figure S7

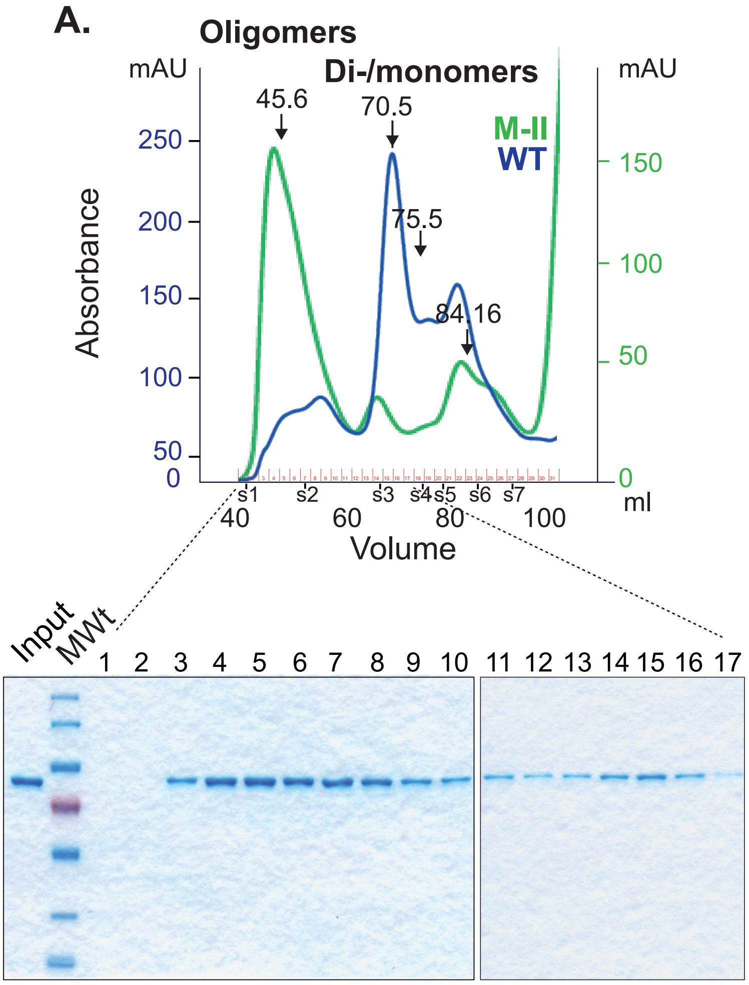

A.
Oligomers
Di-/monomers
mAU
45.6
70.5
75.5
84.16
M-II
WT
Absorbance
Volume
ml
Input
MWt
1 2 3 4 5 6 7 8 9 10 11 12 13 14 15 16 17

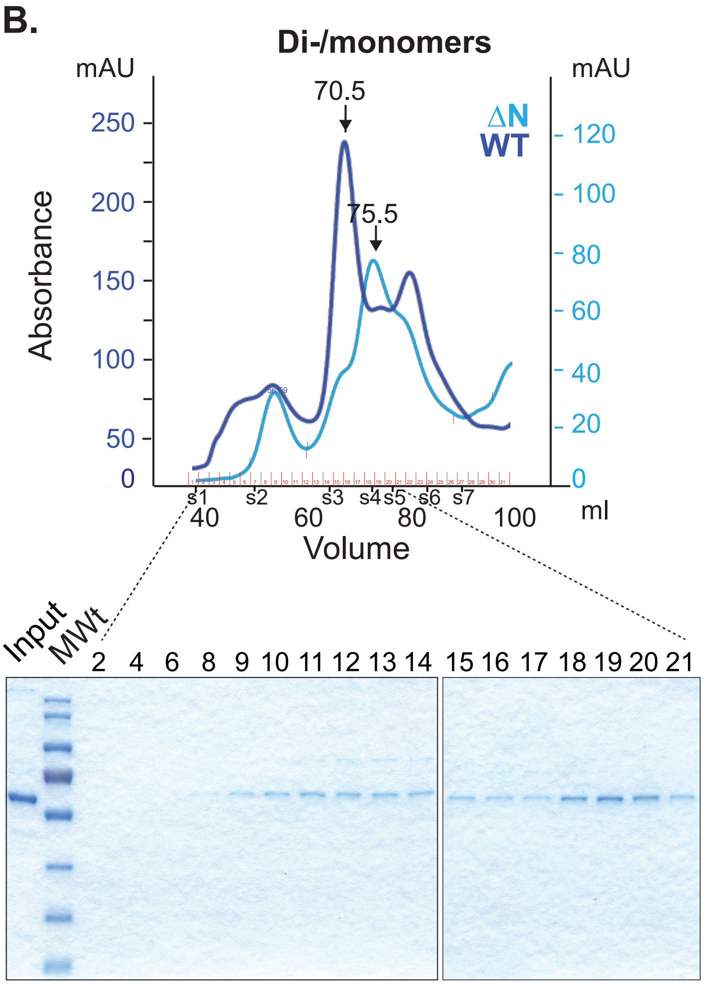

B.
Di-/monomers
mAU
70.5
75.5
ΔN
WT
Absorbance
Volume
ml
Input
MWt
2 4 6 8 9 10 11 12 13 14 15 16 17 18 19 20 21

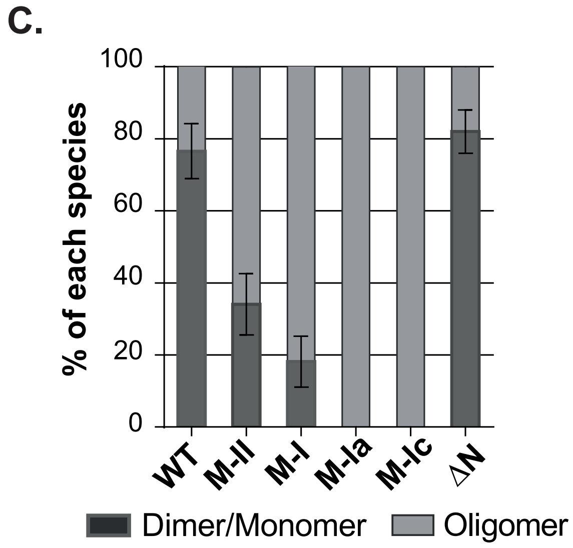

C.
% of each species
WT
M-II
M-I
M-Ia
M-Ic
ΔN
Dimer/Monomer
Oligomer

Figure S8

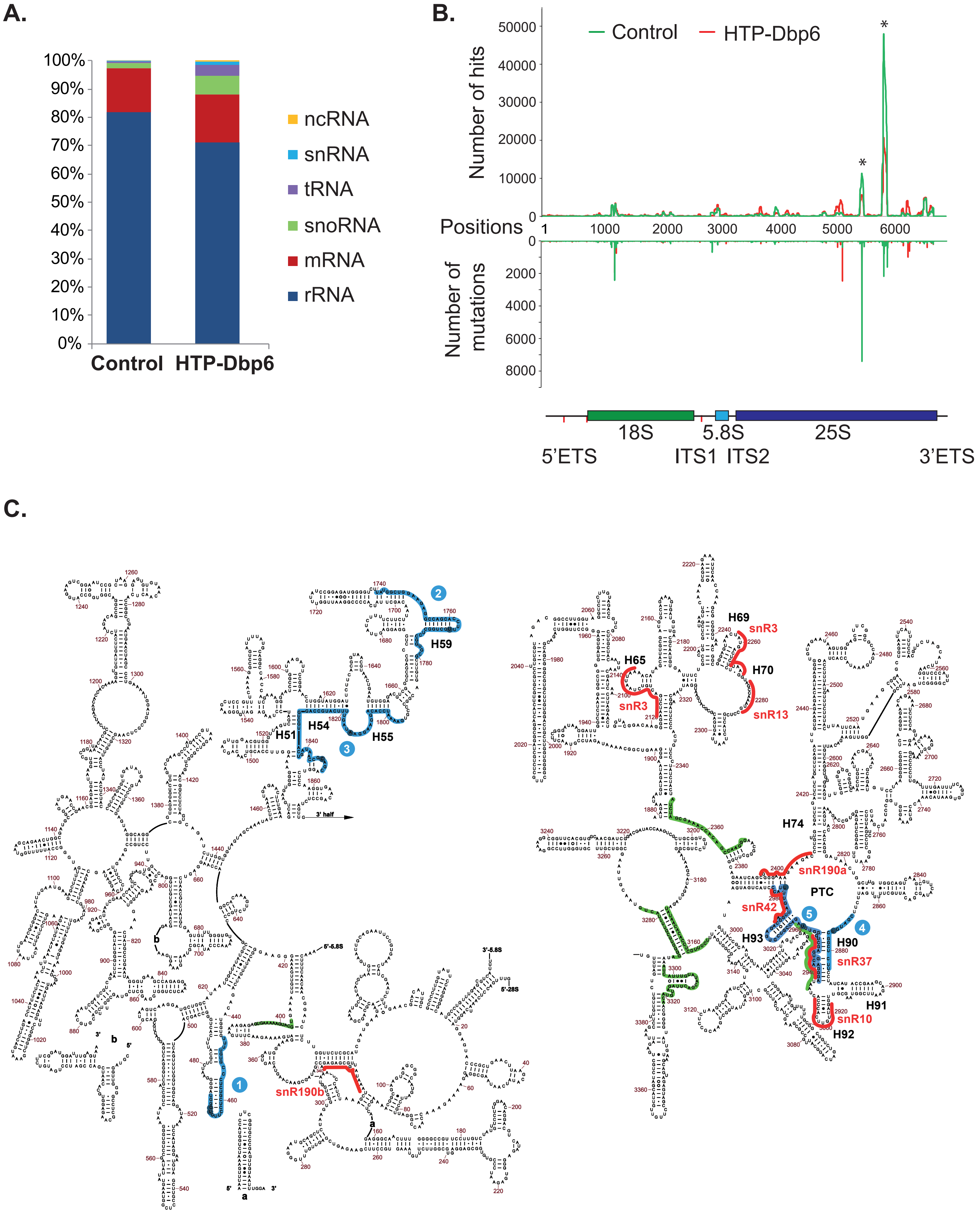
A.
100%
90%
80%
70%
60%
50%
40%
30%
20%
10%
0%
Control
HTP-Dbp6
ncRNA
snRNA
tRNA
snoRNA
mRNA
rRNA
B.
Control
HTP-Dbp6
Number of hits
50000
40000
30000
20000
10000
0
Positions
1
1000
2000
3000
4000
5000
6000
Number of mutations
2000
4000
6000
8000
18S
5.8S
25S
5'ETS
ITS1
ITS2
3'ETS
C.
H59
H54
H55
H51
snR190b
H69
snR3
H65
H70
snR13
H74
snR190a
PTC
snR42
H93
H90
snR37
H91
snR10
H92
3' half
5'-5.8S
3'-5.8S
5'-28S

Figure S9

A.

snoRNA	control	HTP-Dbp6	Fold enrichment / control
SNR30	792	22635	28.57
SNR10	172	4668	27.13
SNR42	151	3854	25.52
SNR3	70	1500	21.42
SNR190	328	5121	15.61
SNR13	92	1269	13.79
SNR37	409	4374	10.69
SNR79	134	1378	10.28
SNR84	84	728	8.66
SNR11	132	822	6.22
SNR60	674	4152	6.16
SNR77	491	1495	3.04
SNR52	1590	4642	2.91
SNR39	307	896	2.91
SNR71	400	1070	2.67

B.

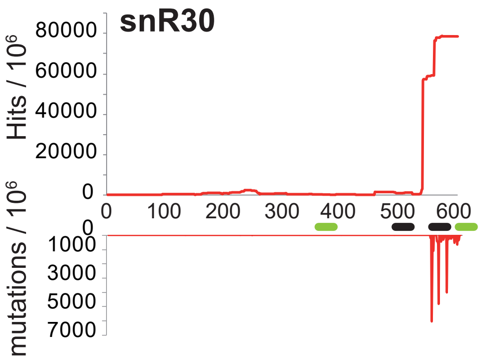

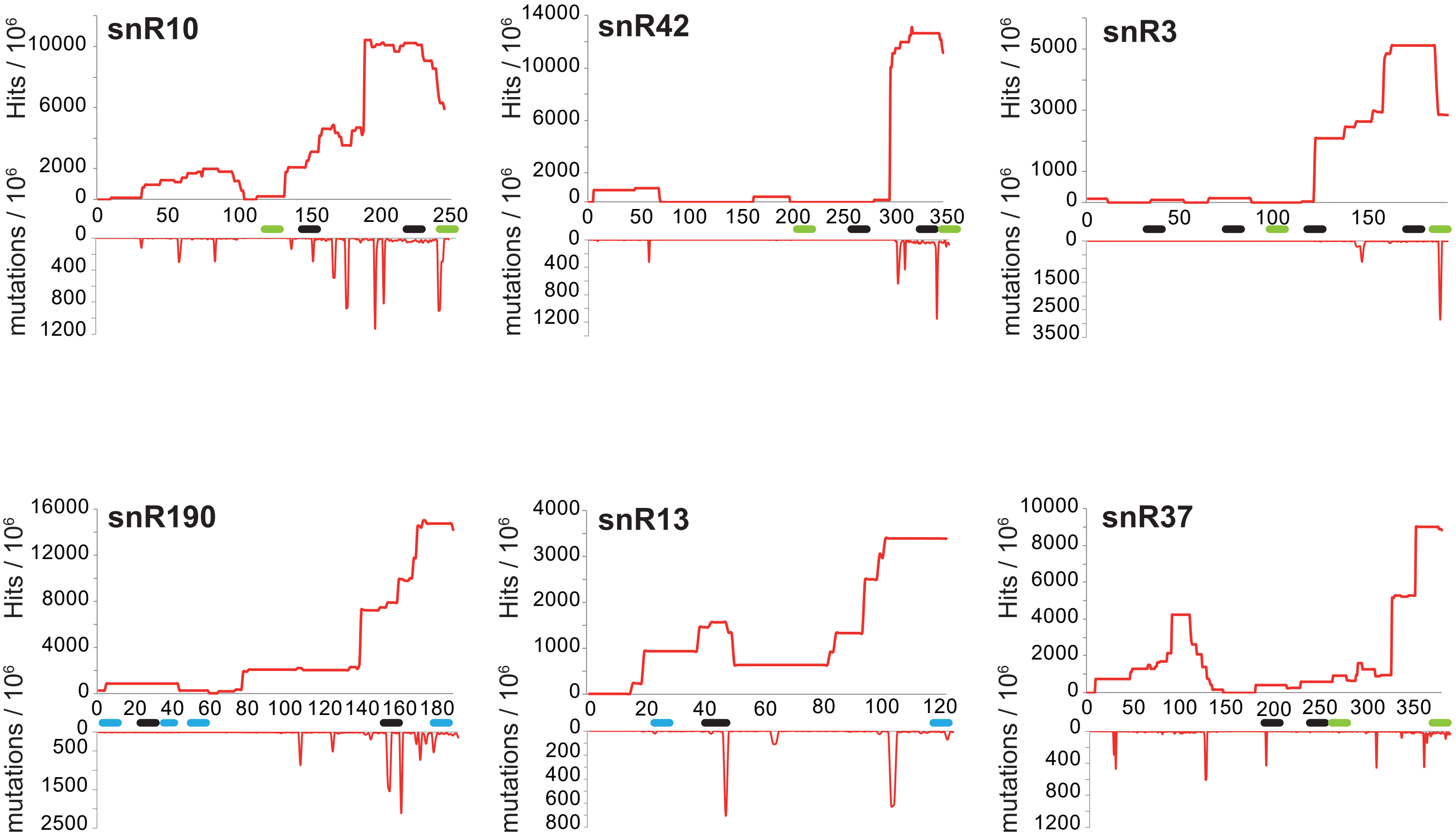

Supplementary table S1

	FORWARD primer 5'-3'	REVERSE primer 5'-3'
WT-Dbp6	CTTTACTTCCAGGGCCATATGTTTGCATCGAGATTTGACCCTAG	TCTAGACTATTAGGATCCTTAAGCTTGTGCGGTGTTATTGTG
ΔN-Dbp6	TCACCAACTAAAACAGATTCCTCAACCTGAATTCGT	TGTTTTAGTTGGTGACCGACGATTCCTTCTGGTGCG
MI-Dbp6	GGAATGGAGTAAGCCAACGTTGCACCTGAACCGGTAGCAG	CTGCTACCGGTTCAGGTGCAACGTTGGCTTACTCCATTCC
MII-Dbp6	CTGGTTCAAAAGACGATCAGCTGCGTCGATAATCAAAAAC	GTTTTTGATTATCGACGCAGCTGATCGTCTTTTGAACCAG
MIa-Dbp6	CTTGTTTATCAATAGTGCTGTGGCCACGATGATTATAC	GTATAATCATCGTGGCCACAGCACTATTGATAAACCAAG
MIc-Dbp6	GATGATCTACTAATGCAGCAGGTGCAGTGATTAAGATATC	GATATCTTAATCACTGCACCTGCTGCATTAGTAGATCATC

Supplementary table S2

	SEQUENCE
**ss oligonucleotides**	
113 ssRNA	5’-GGGAGACCGGAAUUCAUCAUUCAAUAAACUGAUCUUCCGGAUUACCAUGCUUAAGACAUCACGCCUCCAUAUG UCUAUAUAAAGCGCAAAUGGCUGGAAGUAGACCAAUUCAC-3’
ss12-mer	5’-GUAGCGGUUCUG-3’
ss21-mer	5’-GGGAGACCGGAAUUCAUCAUU-3’
ss38-mer	5’-CAGAACCGCUACACUGAAAUGAUCAAUUCCGGUCUCCC-3’
ss38-mer-rev	5’-GGGAGACCGGAAUUGAUCAUUUCAGUGUAGCGGUUCUG-3’
ss58-mer	5’-GGGAGACCGGAAUUCAUCAUUUCAGUGUAGCGGUUCUGACGUGCAAAUCGAUCGUCGA-3’
ss58-mer-rev	5’-UCGACGAUCGAUUUGCACGUCAGAACCGCUACACUGAAAUGAUGAAUUCCGGUCUCCC-3’
ss30-mer	5’-GUAGCGGUUCUGACGUGCAAAUCGAUCGUC-3’
ss12-mer	5’-CAGAACCGCUAC-3’
ss9-mer	5'-AACCGCUAC-3'
**ds oligonucleotides**	
ds58	5’-GGGAGACCGGAAUUCAUCAUUUCAGUGUAGCGGUUCUGACGUGCAAAUCGAUCGUCGA-3’<br>3’-CCCUCUGGCCUUAAGUAGUAAAGUCACAUCGCCAAGACUGCACGUUUAGCUAGCAGCU-5’
ds38	5’-CAGAACCGCUACACUGAAAUGAUCAAUUCCGGUCUCCC-3’<br>3’-GUCUUGGCGAUGUGACUUUACUAGUUAAGGCCAGAGGG-5’
**Hemi-duplex**	
ds38/58	5’-GGGAGACCGGAAUUCAUCAUUUCAGUGUAGCGGUUCUGACGUGCAAAUCGAUCGUCGA-3’<br>3’-CCCUCUGGCCUUAACUAGUAAAGUCACAUCGCCAAGAC-5’
ds12/30	5’-GUAGCGGUUCUGACGUGCAAAUCGAUCGUCGA-3’<br>3’-CAUCGCCAAGAC-5’
**Trap oligonucleotides**	
Comp-58	5’-CAGAACCGCTACAC-3’
Comp-3’	5’-AATGATGAATTCCGGTCTCCC-3’
Comp-5’	5’-GTGAATTGGTCTACTTCCAGC-3’

Supplementary table S3

Northern Blot DNA probes	
**Oligonucleotides**	**Sequences 5'-3'**
23S.1	GATTGCTCGAATGCCCAAAG
rRNA2.1	GGCCAGCAATTTCAAGTTA
18S	CATGGCTTAATCTTTGAGAC
25S	CCATCTCCGGATAAACC

Supplementary table S4: plasmids directing Dbp6 expression in yeast

Plasmid designation	Parental vector	Promoter	Tag
pWT, pM-I, pM-II, pMIa, pM-Ic, pΔN	pHA113	Gar1	ZZ (C-terminal)
Dbp6-GFP, M-II-GFP	pAG413Gal-cdd-EGFP	Gal1	EGFP (C-terminal)
HTP-Dbp6	pRS415	Met25	His tag-Tev cleavage site-ZZ (N-terminal)
